# Light-induced giant random telegraph noise in CuScP_2_S_6_/MoS_2_ heterostructures and their use in noise resilience image inference

**DOI:** 10.1038/s41467-026-71034-6

**Published:** 2026-04-16

**Authors:** Arpan Ghosh, Dipanjan Sen, Samriddha Ray, Rishikesh T. Nair, Anshul Rasyotra, Rui Gusmao, Zdenek Sofer, Saptarshi Das

**Affiliations:** 1 Engineering Science and Mechanics, Penn State University, University Park, PA USA; 2 Department of Inorganic Chemistry, University of Chemistry and Technology, Prague, Czechia; 3 Materials Science and Engineering, Penn State University, University Park, PA USA; 4 Electrical Engineering, Penn State University, University Park, PA USA

**Keywords:** Two-dimensional materials, Electronic devices

## Abstract

Random telegraph noise (RTN) is usually regarded as a hallmark of nanoscale conduction channels, arising from individual trapping events in semiconductors and oxide dielectrics. Here we show that optical excitation can induce “giant” RTN in macroscopically large-area devices based on CuScP_2_S_6_/MoS_2_ heterostructures, revealing a mesoscopic regime in which a sparse set of photo-activated defects in an insulating thiophosphate controls the conductance of an extended channel. Under optical illumination, the device conductance exhibits stochastic two-level fluctuations whose amplitudes are nearly independent of illumination strength, whereas the characteristic trapping-detrapping time constants are strongly governed by the incident light intensity. This behavior implies that photons are absorbed in effectively small packets that modulate a sparse ensemble of active traps, giving rise to bimodal noise statistics and illumination-tunable switching kinetics. We further exploit this controllable stochasticity in a proof-of-concept optical encoder that converts image pixels into RTN-driven spike trains, enhancing the robustness of a spiking neural network (SNN) to noise-corrupted MNIST inputs. Our results identify CuScP_2_S_6_ as a model platform in which light-tunable RTN connects microscopic defect dynamics to macroscopic conductance fluctuations, opening opportunities to engineer noise itself as a functional degree of freedom in photonic and neuromorphic hardware.

## Abstract (web summary)

Random telegraph noise (RTN) reveals charge trapping dynamics in nanoscale electronic devices. Here, authors demonstrate optically controlled RTN in a CuScP2S6/MoS₂ heterostructure, enabling field and light-tunable defect activity and noise resilient neuromorphic image encoding.

## Introduction

Defects occupy a paradoxical role in solid-state devices, where they can act as both performance-limiting elements and functional enablers. While defect engineering has unlocked transformative advances in neuromorphic computing^1–3^, energy harvesting^4^, the microelectronics industry has traditionally pursued defect minimization to ensure high yield, reliability, and scalability. In electronic devices, defects often manifest through charge trapping, giving rise to flicker (1/*f*) noise^5–7^ on short timescales and threshold-voltage drift over longer times due to trapping kinetics. As device dimensions shrink to the deep nanoscale, the statistical influence of individual traps becomes increasingly prominent, causing 1/*f* noise to discretize into random telegraph noise (RTN)^7^, a hallmark of ultra-scaled devices in which only a handful of defects reside within the active region. RTN has been widely employed as a sensitive probe in quantum systems, such as in Elzerman readout schemes and in evaluating the fidelity of spin qubits^8^. In spin-qubit architectures, RTN typically arises from the interaction of a single electron with an isolated atomic-scale defect or quantum dot, enabling the detection of discrete single-charge events with high fidelity due to the well-confined and coherent nature of the quantum system^9,10^. In contrast, RTN in FETs originates from the stochastic trapping and de-trapping of carriers in defect states located within the semiconductor channel or at dielectric interfaces during charge transport. RTN has been almost exclusively treated as an undesirable parasitic effect, particularly in aggressively scaled CMOS devices. By contrast, light-induced RTN is rarely observed, because optical excitation typically generates carriers in extended regions and across many defects, so the resulting conductance changes are spatially and temporally averaged into smooth photocurrent rather than resolvable single-defect switching.

Only a handful of previous studies have demonstrated light-activated RTN. In single-electron transistors (photo SETs)^11^ and nanowire devices^12^, photons modulate remote trap occupancies in oxides or substrates, altering the electrostatic environment and inducing RTN even when traps lie outside the conduction path. A different regime is observed in superconducting resonators under two-photon excitation^13^, where bistable quasiparticle dynamics give rise to phase telegraph noise, emphasizing the role of nonlinear optical processes. These prior observations of photon-modulated RTN are largely confined to ultrasmall or highly specialized structures. To our knowledge, optical RTN has not been reported in mesoscopic devices or in 2D heterostructures, even though the latter have become a central platform for optoelectronics. Moreover, the use of optically driven RTN to directly encode optical signals into stochastic spike trains for neuromorphic inference remains unexplored, despite the potential impact of such in-sensor conversion for vision hardware.

In this work, we uncover a rare and previously unreported phenomenon: the emergence of giant, light-activated RTN in large area CuScP_2_S_6_ (CSPS)-gated monolayer MoS_2_ field-effect transistors (FETs) at sub-ambient temperatures. The high-*κ* CuScP_2_S_6_ dielectric allows strong electrostatic control over the channel MoS_2_ for better current injection. Interestingly, the 2D FETs exhibit RTN-free behavior in the dark, but illumination triggers large-amplitude two-level conductance fluctuations consistent with photon-assisted trapping and detrapping of optically active defect states within the high-*κ* CuScP_2_S_6_. The RTN spike rate increases systematically with incident light intensity, indicating that the dielectric trap kinetics are controlled by the photon flux. We use this behavior to implement an optical encoder in which image pixels are converted into spike trains whose statistics are set by the illumination-dependent RTN. When interfaced with a pre-trained spiking neural network (SNN), this RTN-driven encoding improves robustness to noisy inputs, yielding higher inference accuracies on corrupted MNIST images than a deterministic encoder. These results identify high-*κ* CSPS-gated MoS_2_ FETs as a vdW (van der Waals) platform for light-programmable stochastic neuromorphic hardware based on photon-activated defect dynamics.

## Results

### Device architecture and electrical performance of CuScP_2_S_6_-gated MoS_2_ FETs

**Fig. 1:**
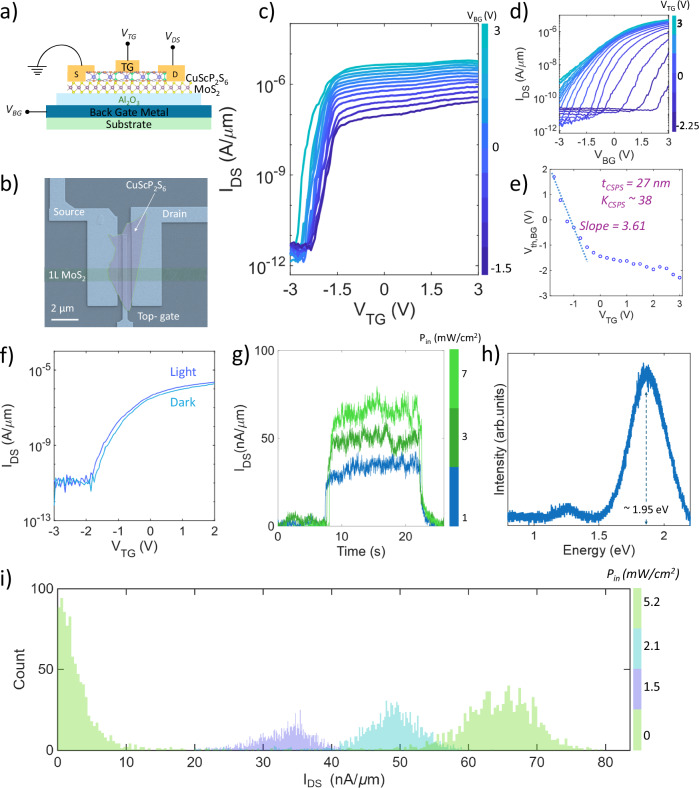
CuScP_2_S_6_ as top-gate dielectric for monolayer MoS_2_ FETs. **a** Schematic and **b** false-colored top scanning electron microscope (SEM) image of a dual-gated 2D FET incorporating CuScP_2_S_6_ as the top-gate dielectric, monolayer MoS_2_ as the semiconducting channel, and 25 nm Al_2_O_3_ as the back-gate dielectric. **c** Top-gate transfer characteristics of MoS_2_ FETs gated with CuScP_2_S_6_ were measured by sweeping the ${{{\rm{V}}}}_{{{\rm{TG}}}}$ for various ${V}_{{\mathrm{BG}}}$ values at a constant ${V}_{{\mathrm{DS}}}$. **d** Back-gate transfer characteristics, i.e., drain-to-source current (${I}_{{\mathrm{DS}}}$) measured by sweeping the back-gate voltage (${V}_{{\mathrm{BG}}}$) for different top-gate voltages (${V}_{{\mathrm{TG}}}$) at a constant drain-to-source voltage, ${V}_{{\mathrm{DS}}}$ = 1 V and **e** back-gate threshold voltage (${V}_{{\mathrm{th}},{\mathrm{BG}}}$) extracted using the iso-current method at ${I}_{{\mathrm{DS}}}$ = 10 nA/µm as a function of ${V}_{{\mathrm{TG}}}$ for CuScP_2_S_6_ gated MoS_2_ FETs. The slope of the ${V}_{{\mathrm{th}},{\mathrm{BG}}}$ versus ${V}_{{\mathrm{TG}}}$ curve at depletion region is proportional to the ratio of the back-gate and top-gate ${\mathrm{EOT}}$ values. The effective dielectric constant was found to be ~38 for a 27 nm thick CuScP_2_S_6_ flake. **f** Top-gate transfer characteristics of representative CuScP_2_S_6_ gated MoS_2_ FETs under dark and maximum illumination intensity at constant ${V}_{{\mathrm{BG}}}$ = 3 V and ${V}_{{\mathrm{DS}}}$ = 1 V. **g** Time-resolved photoresponse (${I}_{{\mathrm{DS}}}$ vs. time) of a representative 2D FET exhibiting positive at various illumination intensities, revealing power-dependent dynamic response. **h** Photoluminescence (PL) spectra of CuScP_2_S_6_ flake. **i** Drain current (*I*_DS_) histogram under dark and three light illuminations (*P*_in_ = 1.5, 2.1, and 5.2 mW/cm^−2^) of the representative 2D FET. The histogram shows the shift of *I*_DS_ under higher illumination and broadening, indicating the presence of stochastic processes in the device.

To investigate the electronic functionality of CSPS as a top-gate dielectric, we fabricated dual-gated MoS_2_ FETs by integrating exfoliated CSPS flakes on top of monolayer MoS_2_ channels. The device schematic and a representative scanning electron microscope (SEM) image are shown in Fig. 1a, b. The device structure consists of a 25 nm Al_2_O_3_ bottom-gate dielectric deposited by atomic layer deposition over a Ti/Pt metal stack, while the top-gate electrode is formed by patterning Ni/Au contacts directly on the exfoliated CSPS flake. The MoS_2_ channel is contacted using Ni/Au source and drain electrodes, creating a vertically stacked vdW heterostructure with CSPS serving as the electrostatically active upper dielectric. Fabrication details can be found in the “Methods” section and in our earlier work^14–17^.

In parallel, extensive material characterization confirms the structural quality of CSPS for device integration. Chemical vapor transport (CVT) grown CSPS crystallizes in a monoclinic *C2/c* structure comprising ${[{{{\rm{P}}}}_{2}{{{\rm{S}}}}_{6}]}^{4-}$ units coordinated by interlayer Cu^+^ and Sc^3+^ cations, consistent with earlier reports^18^ of a ~2.2-2.3 eV insulating bandgap. Atomic force microscopy (AFM) analysis (Supplementary Fig. 1a) shows that the exfoliated CSPS flake possesses a uniform topography with a thickness of approximately 30 nm, as shown in Supplementary Fig. 1b. SEM alongside energy dispersive X-ray (EDS) mapping shown in Supplementary Fig. 2 confirms homogeneous distributions of Cu, Sc, P, and S. Raman spectroscopy (Supplementary Fig. 3) reveals characteristic thiophosphate vibrational modes along with Cu/S lattice features, with subtle spectral shifts suggestive of mild intrinsic disorder. Moreover, the X-ray photoelectron spectroscopy (XPS) spectra (Supplementary Fig. 4) verify the expected Cu⁺, Sc³⁺, and P–S bonding states consistent with the ${[{{{\rm{P}}}}_{2}{{{\rm{S}}}}_{6}]}^{4-}$ framework^18^.The Cu 2p spectrum of CuScP_2_S_6_ exhibits asymmetric broadening arising from plasmon loss features, evident as bump near the Cu 2p_1/2_ peak. For Sc 2p, the reduced 2p_1/2_ peak intensity originates from differential lifetime broadening. Although the 2p_1/2_ peak shows a slightly larger full width at half maximum than 2p_3/2_, the peak area ratio remains within the expected range.

Nevertheless, the back-gate transfer characteristics i.e., the  drain-to-source current (${I}_{{\mathrm{DS}}}$) measured as a function of back-gate voltage (${V}_{{\mathrm{BG}}}$) at ${V}_{{\mathrm{DS}}}$ = 1 V for five representative devices without any applied top-gate (${V}_{{\mathrm{TG}}}$) bias display the expected n-type transport characteristics (Supplementary Fig. 5), consistent with prior reports^17,19–21^. The back gate transfer characteristics for multiple devices form a baseline for transistor functionality and reproducibility. A device-to-device threshold shift is observed due to interface traps and fabrication-related residues at the channel and back gate dielectic interface, i.e., MoS_2_/Al_2_O_3_ interface. Figure 1c presents the top-gate transfer characteristics, i.e., ${I}_{{\mathrm{DS}}}$ versus ${V}_{{\mathrm{TG}}}$ for different ${V}_{{\mathrm{BG}}}$, for a representative CSPS-gated MoS_2_ FET. Top gate transfer characteristics of multiple CSPS gated MoS_2_ FETs show a maximum ON-state current of ~5 μA/μm as depicted in Supplementary Fig. 6. Strong conductance modulation yielding high current on/off ratios and decent subthreshold slopes confirm the effective electrostatic control provided by exfoliated CSPS. It is important to note that in a dual-gated 2D FET architecture, the back gate modulates the entire MoS_2_ channel, including regions beneath the metal contacts, while the top gate modulates only the channel segment between the source and drain. Consequently, ${V}_{{\mathrm{BG}}}$ primarily affects charge injection, whereas ${V}_{{\mathrm{TG}}}$ directly controls channel depletion. Nevertheless, to quantify the dielectric strength of CSPS, we measured back-gate transfer characteristics by stepping the top-gate bias (Fig. 1d) and extracted the corresponding back-gate threshold voltages (${V}_{{\mathrm{th}},{\mathrm{BG}}}$) using an iso-current method at ${I}_{{\mathrm{DS}}}$ = 10 nA/µm. Plotting ${V}_{{\mathrm{th}},{\mathrm{BG}}}$ as a function of ${V}_{{\mathrm{TG}}}$ (Fig. 1e) yields a linear dependence in the depletion regime, and the slope provides the ratio of back-gate to top-gate capacitances, ${C}_{{\mathrm{BG}}}$/ ${C}_{{\mathrm{TG}}}$^22,23^$.$ Using the known ${C}_{{\mathrm{BG}}}$ ≈ 3.3 × 10^−3^ F m^−2^ for 25 nm Al_2_O_3_, we estimate a top-gate capacitance of ${C}_{{\mathrm{TG}}}$ ≈ 9 × 10^−4^ F m^−2^ for the CSPS gate stack. AFM analysis (Supplementary Fig. 7) reveals a typical CSPS flake thickness of ~27 nm, leading to a relative dielectric constant of *κ* ≈ 38, comparable to established high-*κ* transition-metal thiophosphates and oxide dielectrics. Additionally, the top-gate leakage current (${I}_{{\mathrm{TG}}}$) remains below 10^−4^ A cm^−2^ across the entire range of ${V}_{{\mathrm{TG}}}$ (Supplementary Fig. 8), confirming the excellent insulating characteristics and breakdown robustness of exfoliated CSPS. For verifying the temporal stability of CSPS-gated devices, the top gate transfer characteristics of representative CSPS-gated MoS_2_ FETs were measured over 5 consecutive days under identical measurement conditions showing no change in the characteristics, as shown in Supplementary Fig. 9. Collectively, these results establish CSPS as a high-quality, high-*κ* vdW dielectric^24–27^ capable of delivering strong, reliable electrostatic control with low leakage in 2D FET platforms^20^. Additionally, the operational stability of CSPS-gated MoS_2_ FETs was confirmed by repeated top-gate transfer measurements over 10^8^ cycles, as shown in Supplementary Fig. 10, which showed negligible degradation in device characteristics.

To evaluate the optoelectronic functionality of CSPS-gated MoS_2_ FETs, we measured the top-gate transfer characteristics under dark and illuminated conditions. Illumination induces a negative shift in the top-gate threshold voltage (${V}_{{\mathrm{th}},{\mathrm{TG}}}$), revealing photo-induced electrostatic modulation in the CSPS dielectric as shown in Fig. 1f. The corresponding transient photoresponses, measured at ${V}_{{\mathrm{TG}}}$ = 0 V (Fig. 1g), show that the drain current increases during illumination and returns to pre-illumination level when the illumination is turned off. We have used light emtting diodes to invoke responses  rather than LASER illumination to make it a realistic condition for hardware vision^28^. Increasing the illumination intensity (${P}_{{\mathrm{in}}}$) leads to a monotonic rise in photocurrent. We also verified that the photoresponse originates in the CSPS dielctric and not from the MoS_2_ channel, even though MoS_2_ can be photoactive under strong illumination. Supplementary Fig. 11 shows transfer curves of control MoS_2_ transistors with only a back gate; when illuminated at the low illumination level used here, these devices exhibit no measurable photoresponse, ruling out a MoS_2_-intrinsic origin^29,30^. In contrast, photoluminescence (PL) measurements on CSPS exhibit a pronounced emission peak at ~1.95 eV (Fig. 1h), representing a clear redshift relative to the reported bandgap of 2.2-2.3 eV. This PL redshift indicates the presence of defect-related sub-bandgap states that radiatively recombine at lower energies, consistent with a defect-mediated optical response capable of absorbing the low-energy illumination used in our experiments.^24,27^ Therefore, in our devices, the primary optical absorption occurs in the CSPS gate dielectric rather than in the MoS_2_ channel.

Photons excite carriers into mid-gap states in CSPS, changing their charge state and thereby modifying the net fixed charge within the gate stack. This photo-induced charge density $\Delta {Q}_{{\mathrm{CSPS}}}$ acts electrostatically in the same way as an additional gate bias, and therefore manifests in the MoS_2_ channel as a reversible shift in the effective top-gate threshold voltage,

$$\Delta {V}_{{\mathrm{th}},{\mathrm{TG}}}\approx -\frac{\Delta {Q}_{{\mathrm{CSPS}}}}{{C}_{{\mathrm{TG}}}},$$

Under constant ${V}_{{\mathrm{TG}}}$, illumination-driven charging of CSPS defects gradually shifts ${V}_{{\mathrm{th}},{\mathrm{TG}}}$, increasing the channel conductance and producing the observed transient photocurrent. When the light is switched off, the photo-excited defect population relaxes back toward its dark equilibrium via trapping/detrapping and recombination, $\Delta {Q}_{{\mathrm{CSPS}}}$ and ${V}_{{\mathrm{th}},{\mathrm{TG}}}$ decays and hence the drain current return to their pre-illumination values. Thus, the transient photoresponse in the MoS_2_ channel is a direct consequence of reversible, light-induced threshold-voltage modulation originating from charge dynamics in the CSPS gate dielectric. In this picture, MoS_2_ functions primarily as an electrostatic readout channel, rather than the photo absorber.

We also make a key observation: the noise in ${I}_{{\mathrm{DS}}}$ becomes markedly larger under illumination than in the dark. As seen from the drain-current histograms in Fig. 1i, illumination not only shifts the mean current but also broadens the distribution, yielding a higher coefficient of variation ($\sigma /\mu$) than in the dark state. This behavior is not expected from a simple, static threshold-voltage shift, which would primarily change the mean current while leaving the relative fluctuations largely unchanged. Instead, the enhanced coefficient of variation indicates that illumination activates additional stochastic processes in the device.

### Observation of light-induced RTNs in CSPS/MoS_2_ heterostructures

**Fig. 2:**
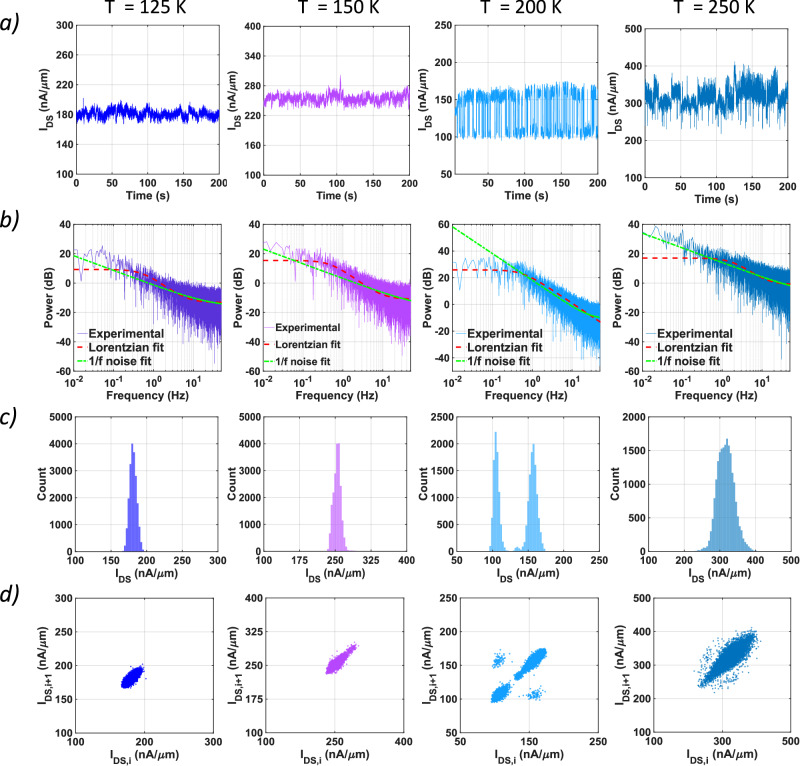
Observation of light-induced RTN in CuScP_2_S_6_-gated MoS_2_ FETs. **a** Time-resolved *I*_DS_ traces recorded at 125, 150, 200, and 250 K under continuous illumination (*P*_in_ = 0.9 mW/cm^2^), exhibiting pronounced RTN with clear step-like transitions, are observed only at 200 K, whereas the traces at other temperatures are comparatively smooth and lack well-defined telegraph switching. **b** Corresponding power spectral density (PSD) spectra obtained via fast Fourier transform (FFT) of the *I*_DS_ fluctuations. A clear enhancement of low-frequency noise at 200 K indicates the onset of RTN. **c** Histogram of *I*_DS_ at 200 K, exhibiting two distinct Gaussian peaks associated with the discrete high- and low-current states of the RTN process. **d** Time-lag plot (TLP) of the *I*_DS_ at 200 K, revealing two well-separated clusters that further confirm the bistable nature of the RTN switching dynamics.

Next, to probe light-induced stochasticity in CSPS/MoS_2_ heterostructures, we performed low-temperature transport measurements under controlled illumination (${P}_{{\mathrm{in}}}$ = 0.9 mW/cm^2^). Lowering the temperature suppresses thermal broadening, smoothens background noise, and slows defect kinetics into the timescale of our measurement bandwidth, so that individual trapping-detrapping events can emerge as RTN rather than being averaged into $1/f$ noise^31–33^. Figure 2a, b, respectively, compares the time traces of ${I}_{{\mathrm{DS}}}$ and corresponding fast Fourier transforms (FFTs) at $T$ = 125, 150, 200 and 250 K. Multiple devices show RTN under optical stimuli for different temperatures (Supplementary Fig. 12). Unlike nonvolatile photo-induced memory, RTN is stochastic and do not retain at particular state for longer duration once the external stimulus (illumination or bias) is removed. The random trapping-detrapping does not allow any retention of the states in RTN. Pronounced RTN with clear step-like transitions is observed only at 200 K, whereas the traces at other temperatures are comparatively smooth and lack well-defined telegraph switching. Likewise, a distinct ${1/f}^{2}$-type Lorentzian spectrum is observed at 200 K, confirming bistable RTN dynamics in the frequency domain, while the other temperatures are dominated by featureless $1/f$-like noise.

Across the full temperature series, the discrete nature of the fluctuations is corroborated by the ${I}_{{\mathrm{DS}}}$ histograms and time-lag plots (TLPs) in Fig. 2c, d. At 200 K, where RTN is visible, the histograms show two well-separated peaks associated with high- and low-conductance states, while the corresponding TLP, constructed by plotting ${I}_{{\mathrm{DS}}}(i)$ versus ${I}_{{\mathrm{DS}}}(i+1)$ display two elongated clusters associated with persistence in each state, together with a substantial population of off-diagonal points arising from frequent state-to-state transitions. This fingerprint is characteristic of a Markovian two-state process governed by a small number of dominant traps, rather than broadband conductance noise. The non-monotonic temperature dependence is consistent with a thermally activated mechanism: at higher temperatures (250 K), the trap kinetics are too fast and average out, while at lower temperatures (150 and 125 K), the traps are effectively frozen in one charge state over the measurement window. At the intermediate temperature of 200 K, the trapping and detrapping times become comparable to the integration time, yielding resolvable two-level fluctuations. Strikingly, the RTN amplitude in this regime is giant, with current steps on the order of ~60 nA, indicating that photoactivated charge rearrangements in the CSPS dielectric produce large, discrete shifts in the effective top-gate potential and strongly modulate the MoS_2_ channel conductance.

**Fig. 3:**
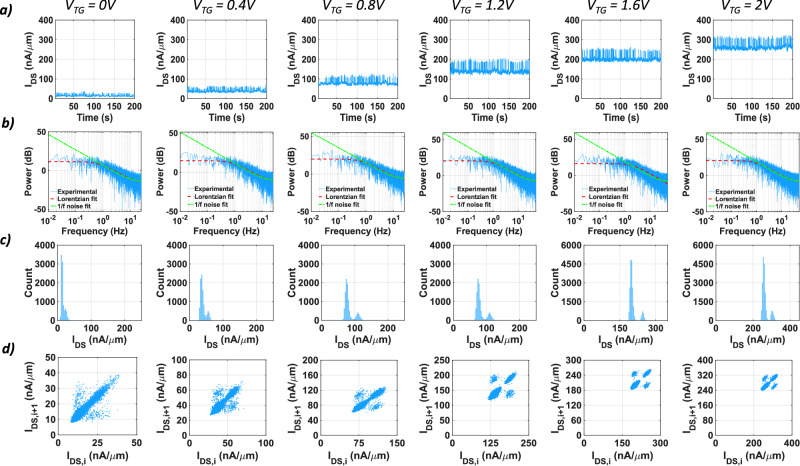
Gate-bias-dependent evolution of light-induced RTN in CuScP_2_S_6_/MoS_2_ heterostructure. **a** Time-resolved *I*_DS_ traces recorded at 200 K under continuous illumination (*P*_in_ = 0.9 mW/cm^2^) for different top-gate voltages (*V*_TG_), revealing frequent transitions among multiple *I*_DS_ levels, indicating the participation of several photoactive traps with comparable electrostatic influence. At higher *V*_TG_ values, these multi-level fluctuations progressively collapse into a well-defined two-level telegraph signal, dominated by a single trap. **b** Corresponding power spectral density (PSD) spectra obtained via fast Fourier transform (FFT) of the *I*_DS_ fluctuations, the PSD broadens at higher *V*_TG_ values due to enhanced intra-level fluctuations. **c** Histograms of *I*_DS_ at different *V*_TG_, revealing the same evolution: broad, multi-peak distributions at low *V*_TG_; clean, bimodal peaks corresponding to two RTN states at intermediate *V*_TG_; and finally broadened peaks as intra-level noise increases at high *V*_TG_. **d** Time-lag plots (TLPs) showing multi-cluster patterns at low *V*_TG_, two well-separated elongated clusters when the RTN reduces to a two-level process, and thicker clusters at high *V*_TG_ reflecting increased noise within each state.

Next, we found that the carrier concentration in the MoS_2_ channel tuned by the top-gate voltage, strongly modulates both the character and magnitude of the light-induced RTN. Under fixed illumination (${P}_{{\mathrm{in}}}$ = 0.9 mW/cm^2^) at 200 K, the ${I}_{{\mathrm{DS}}}$ time traces in Fig. 3a show a clear evolution with ${V}_{{\mathrm{TG}}}$. At low ${V}_{{\mathrm{TG}}}$(0-0.4 V), where the channel is weakly accumulated, the fluctuations are small (∼20 nA) and involve frequent transitions among multiple close conductance levels, indicating several photoactive traps with comparable electrostatic leverage. As ${V}_{{\mathrm{TG}}}$ is increased (0.8-1.2 V), the multi-level behavior progressively collapses into a dominant two-level telegraph signal, and the RTN step size grows markedly, reaching a maximum of ∼60 nA near ${V}_{{\mathrm{TG}}}$ = 1.2 V. At even higher accumulation (1.6-2 V), the two states remain discernible, but the plateaus become noticeably noisy, and the discrete step size decreases modestly (∼50 nA), suggesting reduced effective trap leverage due to channel screening and/or a reduction in ${g}_{{{\rm{m}}}}$ at strong accumulation. The spectral and statistical analyses in Fig. 3b–d corroborate this gate-driven crossover. The FFT power spectra (Fig. 3b) are well captured by a Lorentzian component superimposed on a $1/f$ background, confirming RTN-dominated noise where switching is resolvable. Consequently, the histograms (Fig. 3c) evolve from broadened, weakly resolved distributions at low ${V}_{{\mathrm{TG}}}$ to clear bimodal peaks at intermediate ${V}_{{\mathrm{TG}}}$, and then to broadened bimodal peaks at high ${V}_{{\mathrm{TG}}}$ as intra-state noise increases. The time-lag plots (Fig. 3d) show the same trend: diffuse, near-diagonal clouds at low ${V}_{{\mathrm{TG}}}$ reflecting rapid multi-state hopping; two elongated lobes with substantial off-diagonal transition density at intermediate ${V}_{{\mathrm{TG}}}$ characteristic of a Markovian two-state process; and thicker lobes at high ${V}_{{\mathrm{TG}}}$ consistent with persistent two-state RTN accompanied by enhanced intra-level fluctuations. The high amplitude RTN of the order of tens of nanoamperes in the 2D channel implies the strong electrostatic control on the trap available in the gate dielectric, CuScP_2_S_6_, rather than the MoS_2_ channel.

### Control of RTN kinetics and spike statistics through illumination strength

**Fig. 4:**
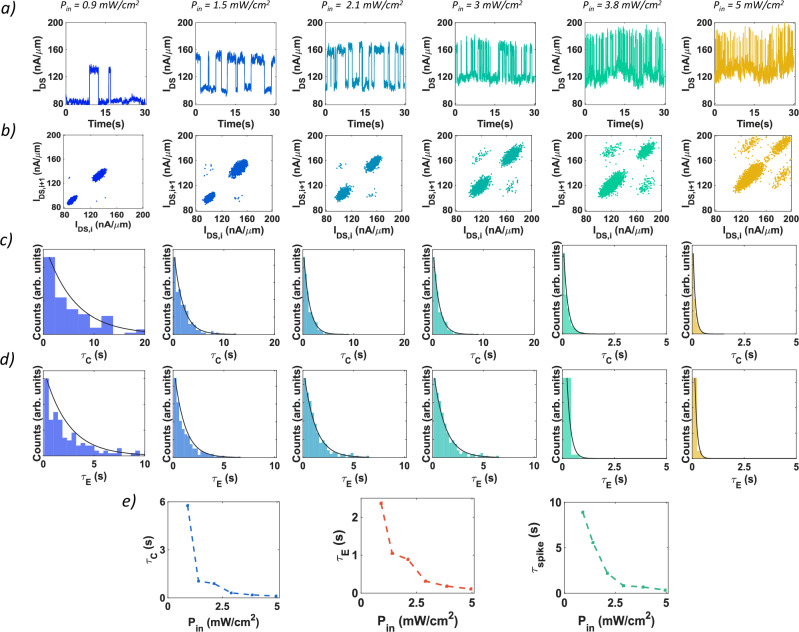
Illumination-dependent modulation of RTN dynamics in CuScP_2_S_6_-gated MoS₂ FETs. **a** Time-resolved ${I}_{{\mathrm{DS}}}$ traces recorded under increasing illumination power, ${P}_{{\mathrm{in}}}.$ At low ${P}_{{\mathrm{in}}}$, the device exhibits slow, long-lived two-level switching with well-separated plateaus. As ${P}_{{\mathrm{in}}}$ increases, the dwell times shorten and switching accelerates, producing denser RTN spikes while maintaining clear contrast between the two dominant ${I}_{{\mathrm{DS}}}$ states. **b** Time-lag plots (TLPs) corresponding to the traces in (**a**), each retaining the characteristic bimodal clustering of a two-state fluctuator. With increasing ${P}_{{\mathrm{in}}}$, the clusters broaden and partially overlap, reflecting increased variability in switching dynamics while still preserving a bistable structure. **c**, **d** Extracted distributions of capture $({\tau }_{c})$ and emission $({\tau }_{e})$ dwell times obtained using a Canny-edge-based step-detection algorithm applied to the ${I}_{{\mathrm{DS}}}\left(t\right)$ trajectories. **e** Illumination-dependent evolution of the average capture time ($\bar{{\tau }_{c}}$), emission time ($\bar{{\tau }_{e}}$), and inter-spike interval ($\bar{{\tau }_{{spike}}}$), demonstrating that higher photon flux accelerates both capture and emission processes.

We next examined how illumination strength modulates the RTN dynamics in CSPS-gated MoS_2_ FETs at 200 K. Figure 4a shows ${I}_{{\mathrm{DS}}}(t)$ traces under increasing incident power ${P}_{{\mathrm{in}}}$ under a constant back gate (*V*_BG_) and top gate (*V*_TG_) voltages. At low illumination (${P}_{{\mathrm{in}}}$ = 0.9 mW/cm^2^), the current exhibits slow, long-lived two-level switching: the RTN states are well separated and the device dwells for several seconds in each level before switching. As ${P}_{{\mathrm{in}}}$ is increased, the RTN states become progressively shorter-lived, and the switching accelerates: spikes appear more densely in time, and the dwell intervals shrink, while the contrast between the high- and low-${I}_{{{\rm{DS}}}}$ levels remains clearly visible. The TLPs in Fig. 4b makes this observation especially clear. Each TLP retains the bimodal fingerprint of a two-state process, with two clusters corresponding to the low- and high-current states. Importantly, the density of off-diagonal points increases systematically with ${P}_{{\mathrm{in}}}$, directly indicating a larger number of state-to-state transitions occurring between adjacent samples. Thus, higher illumination enhances the trap switching kinetics on the measurement timescale. While the clusters broaden somewhat at high ${P}_{{\mathrm{in}}}$ (reflecting increased timing variability and added intra-state noise), their continued separation confirms that the dynamics remain governed by a small set of bistable, photoactivated traps rather than evolving into broadband, featureless noise.

To quantify the defect kinetics, we extracted the distribution of capture time (${\tau }_{c}$) and emission time (${\tau }_{e}$) from the RTN traces as a function of ${P}_{{\mathrm{in}}}$, as shown in Fig. 4c, d. A Canny-edge–based step-detection algorithm^34^ was applied to the ${I}_{{\mathrm{DS}}}(t)$ trajectories to identify sharp upward and downward steps corresponding to capture and emission events. The dwell times in the low- and high-current states were then compiled from these events, focusing on the dominant two-level component and disregarding occasional weak metastable intermediates. This two-state approximation enables fitting with single-exponential decays to extract the average capture and emission time constants, $\bar{{\tau }_{c}}$ and $\bar{{\tau }_{e}}$, at each ${P}_{{\mathrm{in}}}$, consistent with standard two-state Markov kinetics. Figure 4e summarizes the illumination dependence of $\bar{{\tau }_{c}}$, $\bar{{\tau }_{e}}$, and the inter-spike interval ($\overline{{\tau }_{{spike}}}$). At the lowest illumination, ${P}_{{\mathrm{in}}}$ = 0.9 mW/cm^2^, both $\bar{{\tau }_{c}}$ and $\bar{{\tau }_{e}}$ are on the order of several seconds (e.g., $\bar{{\tau }_{c}}$ ≈ 5.8 s), and the $\overline{{\tau }_{{spike}}}$ is similarly long, reflecting slow transitions across large relaxation barriers that cannot be overcome thermally at 200 K. As ${P}_{{\mathrm{in}}}$ increases, $\bar{{\tau }_{c}}$, $\bar{{\tau }_{e}}$, and $\overline{{\tau }_{{spike}}}$ all decrease monotonically, reaching ~ 0.1 s at ${P}_{{\mathrm{in}}}$ = 6 mW/cm^2^. Thus, higher photon flux makes the RTN states both more frequent and more short-lived. This trend is consistent with a photon-assisted non-radiative multiphonon (NRMP) mechanism^35,36^, in which optical excitation increases the rate of carrier excitation into and out of deep defect levels and effectively lowers the activation barrier for both capture and emission. The close tracking between $\bar{{\tau }_{c}}$, $\bar{{\tau }_{e}}$, and $\overline{{\tau }_{{spike}}}$ directly links the microscopic defect kinetics to the emergent spike statistics, demonstrating that the spike rate, and hence the stochastic encoding of optical intensity, can be continuously tuned by the incident photon flux.

As mentioned earlier, a striking feature of the CSPS/MoS_2_ heterostructure is the unusually large RTN amplitude, which reaches ~50 nA even at the lowest illumination and increases to ~80 nA under higher photon flux, as shown in Supplementary Fig. 13. This “giant” modulation arises from strong electrostatic coupling between deep, localized defects in CSPS and the atomically thin MoS_2_ channel. When a defect near the interface changes its charge state upon absorbing photons, it produces a substantial local potential shift; because conduction in monolayer MoS_2_ is tightly confined to this interface, a single trapping or detrapping event can alter the channel conductance significantly. Increasing illumination enhances the completeness and frequency of these charge-state transitions, further amplifying the effective step height.

### Stochastic spike encoding for noise resilient neuromorphic inference

**Fig. 5:**
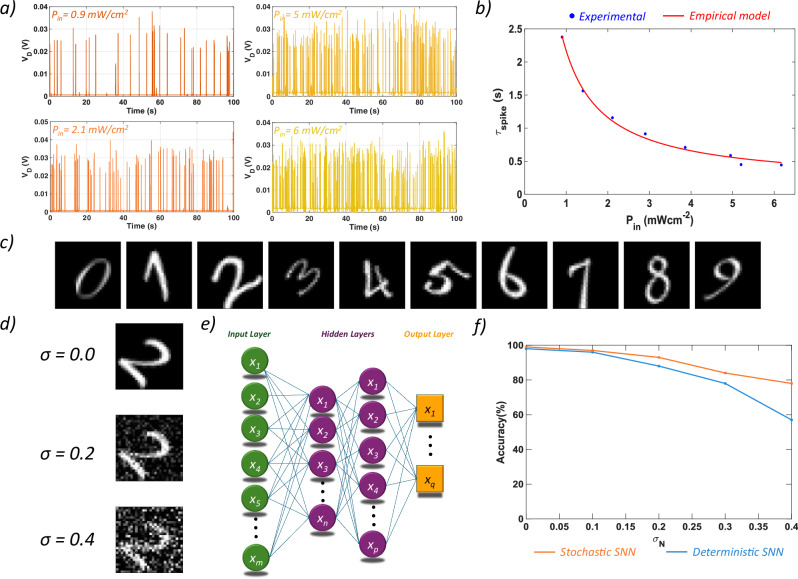
Light-induced RTN for stochastic encoding. **a** Representative time-domain voltage (${V}_{{{\rm{D}}}}$) traces showing the RTN-induced spike trains for four input power levels, where higher ${P}_{{\mathrm{in}}}$ produces shorter $\bar{{\tau }_{{spike}}}.$ **b** Exponential fit derived from experimental measurements demonstrating the inverse relationship between the mean inter-spike interval $\bar{{\tau }_{{spike}}}$ and incident optical power ${P}_{{\mathrm{in}}}$, reflecting the stochastic defect-mediated dynamics of the device. **c** Ten MNIST digits utilized to train the baseline artificial neural network (ANN). **d** Noisy input images generated by adding Gaussian perturbations, used to evaluate the robustness of the ANN-to-SNN converted network. **e** Schematic of the feedforward neural network used in this work, which receives image pixels and outputs one of the ten-digit classes. **f** Classification accuracy of the spiking neural network under varying noise levels (*σ* = 0-0.4), highlighting that the stochastic photon-induced RTN encoder consistently outperforms deterministic encoding schemes.

Having established that photon flux continuously tunes the RTN switching kinetics and inter-spike intervals in CSPS/MoS_2_ FETs, we next investigate whether this illumination-governed stochasticity can be harnessed for noise-resilient neuromorphic inference drawing inspiration from the working of the biological brain as shown in Fig. 5. Stochastic spike encoding is theoretically known to improve robustness, uncertainty tolerance, and generalization in neuromorphic systems because information is carried in spike distributions rather than in single, precise spike times^37,38^. However, there is currently no hardware platform that directly converts optical intensity into physically generated stochastic spike trains with tunable statistics; most existing encoders rely on deterministic mappings (e.g., rate or time-to-first-spike encoding) or on algorithmic pseudo-randomness layered on top of conventional sensors^39,40^. Our photon-activated RTN in CSPS/MoS_2_ heterostructures fills precisely this gap. In our implementation, the key encoding variable is the $\overline{{\tau }_{{spike}}}$, which emerges directly from the illumination-controlled RTN kinetics.

We operated the device in voltage mode, i.e., we measured the drain voltage while injecting a constant drain current. In this configuration, the RTN dynamics no longer appear as two comparable long-lived current states; instead, one state becomes long-lived while the other is short-lived, producing isolated, spike-like voltage excursions as show in Fig. 5a. Note that voltage spikes are naturally suited for neuromorphic encoding and direct interfacing with peripheral circuits. This representation also provides a clearer, hardware-level definition of $\overline{{\tau }_{{spike}}}$, defined as the time between successive short-lived excursions from the baseline state. Even in this voltage-mode operation, $\overline{{\tau }_{{spike}}}$ retains a monotonic dependence on ${P}_{{\mathrm{in}}}$, decreasing systematically with increasing illumination (Supplementary Fig. 14). We quantitatively modeled this behavior across the full range of ${P}_{{\mathrm{in}}}$ used in experiments, obtaining a compact description of how photon flux sets the spike timing statistics:

1 $$\overline{{\tau }_{{spike}}}={\tau }_{0}\left(\frac{{P}_{0}}{{P}_{{\mathrm{in}}}}+1\right)$$

where ${P}_{0}$ and ${\tau }_{0}$ are fitting parameters extracted from device measurements as shown in Fig. 5b. This model serves as a bridge between experiment and simulation, enabling us to generate realistic, illumination-tunable stochastic spike trains for all 784 pixels of each Modified National Institute of Standards and Technology (MNIST) digit (digits 0–9, each 28 × 28 pixels). Figure 5c illustrates 10 MNIST images.

First, we trained a baseline artificial neural network (ANN) for classification of the MNIST digits and then converted it to a SNN following the near-lossless ANN–SNN conversion scheme^38,41^. The ANN consists of 784 input neurons, 3 fully connected hidden layer with 512, 256, and 128 neurons, respectively, and 10 output neurons corresponding to the ten digits as summarized in Fig. 5e. Each 28 × 28 grayscale image is flattened into a 784 × 1 vector and fed to the input layer. The network is trained using gradient descent with a learning rate of 0.001 for 3 epochs. ReLU is used as the hidden-layer activation function so that its behavior closely matches that of integrate-and-fire neurons in the converted SNN. A total of 50,000 images from the MNIST dataset are used for training, and the training accuracy converges to ~96.4%.

After training, the ANN is converted into an SNN where 1000 images are used for testing. We first implemented a conventional deterministic rate-encoding scheme in which each MNIST pixel intensity (0-255) is linearly mapped to a fixed number of spikes over a 300-timestep window. In this encoding, a pixel value of 0 produces no spikes, whereas a value of 255 yields a spike at every timestep, resulting in a fully saturated spike train. The ANN trained on the MNIST dataset was then converted to an SNN using this deterministic rate map, and the converted SNN achieved an inference accuracy of 98%. In contrast, when pixels were mapped probabilistically, and the ANN was converted to stochastic SNN, yielding an inference accuracy of 99%. Next, to assess robustness against corrupted inputs, we injected additive Gaussian noise with standard deviation ${\sigma }_{N}$ up to 0.4 into the MNIST images. Representative noisy examples are shown in Fig. 5d. Under noisy conditions, deterministic encoding, which maps pixel intensities to fixed spike intervals, suffers a degradation in inference accuracy (from ~98 to ~57%) because corrupted inputs directly distort the spike timing. In contrast, the CSPS-based stochastic encoder remains highly robust, with accuracy decreasing only modestly (from ~99 to ~78%) across the same noise levels (Fig. 5d). This resilience arises because $\overline{{\tau }_{{spike}}}$ distributions carry pixel information probabilistically, allowing noisy pixels to generate overlapping, jitter-tolerant spike patterns rather than deterministic failures. The inherent variability in illumination-dependent $\overline{{\tau }_{{spike}}}$ thus acts as a built-in noise-filtering mechanism, closely mirroring biological sensory systems in which stochastic firing stabilizes perception. These results position photon-induced RTNs in CSPS/MoS_2_ heterostructures as a practical and powerful platform for hardware-level, noise-tolerant neuromorphic encoding, particularly well suited for image processing tasks.

In conclusion, we identify CuScP_2_S_6_ (CSPS) as a high-*κ* van der Waals dielectric that is not only electrostatically effective for monolayer MoS_2_ FETs but also hosts optically active deep defects that reshape the device noise response. Under low-intensity illumination and at sub-ambient temperatures, these devices exhibit giant, light-induced RTN in a mesoscopic geometry, with a well-defined temperature window where photon-assisted trapping-detrapping in CSPS produces large, discrete conductance steps. The RTN kinetics are tunable by both carrier density and photon flux and are consistent with a photon-assisted NRMP mechanism. By operating the device in voltage mode, we show that these defect dynamics naturally generate spike-like voltage excursions whose inter-spike interval depends monotonically on illumination, enabling direct conversion of optical intensity into stochastic spike trains. Using an empirical model calibrated to the measured spike statistics, we implement a hardware-grounded stochastic encoder that drives a pretrained SNN and achieves markedly improved robustness to noisy images compared with deterministic encoders. Our work thus establishes CSPS/MoS_2_ heterostructures as a platform where light-programmable RTN can be harnessed as a functional resource for noise-tolerant neuromorphic vision hardware.

## Methods

### Synthesis of CuScP_2_S_6_ (CSPS) crystals

CuScP_2_S_6_ crystals were grown using the chemical vapor transport (CVT) method in a two-zone furnace. Pre-synthesized polycrystalline CuScP_2_S_6_ materials (corresponding to 12 g) were initially combined with transport agent I_2_ (500 mg) and loaded into a fused silica tube with 16 and 18 mm inner and outer diameters, respectively and a length of 28 cm. The mixture was sealed under vacuum conditions using an oxy/natural gas torch. The ampoules were heated first at 450°C for 25 hours and then at 500°C for 25 hours. Then, the ampoules were heated at 650°C for 100 hours. The ampoules were finally placed in a two-zone CVT furnace. The CVT growth process involved two temperature zones: *source zone (660 °C)*: the mixture was placed in this zone. Initially for 48 hours, the temperature increased to 600 °C. Subsequently, it was heated to 650 °C over 3 h and maintained for 14 days. Finally, the temperature was reduced to ambient levels over 12 h. *Deposition zone (560 °C)*: CuScP_2_S_6_ crystals were obtained in this zone. The heating process followed a similar pattern: 48 hours to 700 °C, 3 h to 600 °C, 144 h holding time, and cooling to ambient temperature over 12 h. Notably, these CuScP_2_S_6_ crystals were collected from the CVT tubes inside the - Argon filled glovebox, as these crystals are highly sensitive to humidity. Furthermore, these crystals are characterized directly without any further processing steps. The resulting crystals exhibit uniform-plane surfaces, making them suitable for device fabrication.

### Large area monolayer MoS_2_ film growth

Monolayer MoS_2_ films were grown using metal organic chemical vapor deposition (MOCVD) on a 2-inch pre-scored, double-side polished c-plane sapphire substrate. To ensure uniform deposition, cold-wall horizontal reactor with an inductively heated graphite susceptor and wafer rotation was employed. As precursors for molybdenum and sulphur, molybdenum hexacarbonyl (Mo(CO)_6_) and hydrogen sulfide (H_2_S) are used, respectively. The metal precursor, Mo(CO)_6_, is maintained at 10 °C and 950 Torr in a stainless-steel bubbler, delivering a flow of 0.036 sccm, while 400 sccm of H_2_S was supplied. The deposition took place at 1000 °C and 50 Torr under a hydrogen atmosphere, achieving monolayer growth in 18 min. Prior to growth, the substrate was preheated to 1000 °C in H_2_ for 10 min. Following deposition, the substrate was cooled in H_2_S to 300 °C to prevent decomposition of the MoS_2_ film. The fully coalesced monolayer MoS_2_ was achieved across 2″ sapphire substrate.

### X-ray photoelectron spectroscopy

XPS measurements were performed using a Thermo Scientific NEXSA G2 XPS equipped with an electron flood gun and a scanning ion gun at room temperature. The crystals were placed on copper tape, which served as the adhesive substrate. The data were analyzed using Thermo Scientific Advantage Data System software.

### Atomic force microscopy

Atomic force microscopy was utilized to investigate the thickness profile of exfoliated multilayer flakes before. RTESPA-150 probe tips were used with a Bruker Dimension Icon AFM. All images were collected in peak force tapping mode with a peak force of 12 nN and a scan rate of 0.5 Hz. Images were processed and exported using Gwyddion.

### Scanning electron microscopy

Scanning electron microscopy (SEM): SEM of the 2D MoS2 transistors used in this study was conducted using a Zeiss Gemini 500 field emission scanning electron microscopy (FESEM) system at an accelerating voltage of 5 kV.

### Energy-dispersive X-ray spectroscopy (EDS)

EDS analysis was performed on micrometer-sized bulk crystal flakes using an ESEM Q250 SEM instrument. A tungsten source with an accelerating voltage of 30 kV was utilized to determine the elemental composition of the crystals.

### Raman and photoluminescence spectroscopy

Raman and PL spectra measurement was acquired on a Horiba LabRAM HR evolution confocal Raman microscope, using a 532 nm laser set to 34 mW, filtered at 1%. The setup included a 100× objective with a numerical aperture of 0.9, and gratings with spacing of 1800 and 300 gr mm^−1^ for Raman and PL, respectively.

### Fabrication of dual-gated 2D FETs

To begin with, monolayer MoS_2_ was transferred on 25 nm thick Al_2_O_3_ substrates. The substrate was spin-coated with PMMA A6 (4000 RPM for 45 s) and baked at 180 °C for 90 s. The resist was then exposed using a Raith EBPG5200 e-beam lithography tool and developed with a 1:1 mixture of 4-methyl-2-pentanone (MIBK) and IPA for 60 s, followed by a 45 s IPA rinse. The exposed monolayer MoS_2_ film was etched using an SF_6_ RIE process at 5 °C for 11 s. Afterward, the sample was cleaned in acetone and IPA to remove the e-beam resist. Next, the 2D dielectrics were mechanically exfoliated on top of the pre-patterned MoS_2_ channels. A second lithography step was performed to form the source-drain and top-gate electrodes. The substrate was spin-coated at 4000 RPM for 45 s with methyl methacrylate (MMA) EL6 and PMMA A3; these resists were baked at 150 and 180 °C for 90 s each, respectively. E-beam lithography was used again to pattern the source and drain, followed by development in MIBK/IPA and an IPA rinse, as before. For MoS_2_ FETs, 50 nm of Ni and 30 nm of Au were deposited via e-beam evaporation to form the electrodes. Finally, a lift-off process was conducted to remove excess Ni/Au by soaking the sample in acetone for 1 h, followed by a 30-min IPA rinse to clean the substrate.

### Electrical characterization

Electrical characterization of the fabricated devices was performed using a Lake Shore CRX-VF probe station under atmospheric conditions and low temperature with a Keysight B1500A parameter analyzer. A continuous wave white light source was used for all experiments involving light illumination unless otherwise stated.

## Supplementary information

Supplementary Information

Transparent Peer Review File

## Data Availability

Datasets generated during and/or analyzed during the current study are available from the corresponding author on request.

## Code availability

The codes used for plotting the data are available from the corresponding author on request.
