# Supplementary material for: Light-induced giant random telegraph noise in CuScP2S6/MoS2 heterostructures and their use in noise resilience image inference
Source: Nat Commun. 2026 Apr 16;17:5297. doi: 10.1038/s41467-026-71034-6 (PMC13270004; doi:10.1038/s41467-026-71034-6)
Supplement: Supplementary file 1 — Supplementary Information [file 41467_2026_71034_MOESM1_ESM.pdf]

Supplementary Information

Light-induced giant random telegraph noise in $CuScP_2S_6/MoS_2$ heterostructures and their use in noise resilience image inference

Arpan Ghosh[1,#], Dipanjan Sen[1,#], Samriddha Ray[1], Rishikesh T. Nair[1], Anshul Rasyotra[1], Rui Gusmao[2], Zdenek Sofer[2], and Saptarshi Das[1,3,4]*

[1]Engineering Science and Mechanics, Penn State University, University Park, PA 16802, USA

[2]Department of Inorganic Chemistry, University of Chemistry and Technology Prague, Prague 16628 Czech Republic

[3]Materials Science and Engineering, Penn State University, University Park, PA 16802, USA

[4]Electrical Engineering, Penn State University, University Park, PA 16802, USA

[#]Equal Contribution

[*] Corresponding Author: email – sud70@psu.edu

Supplementary Figure 1

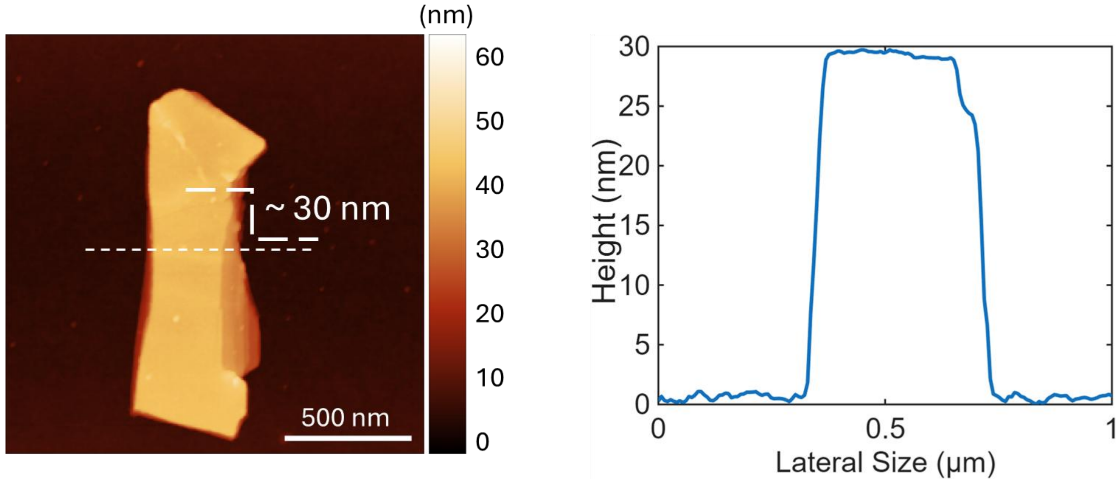

Supplementary Figure 1. *a) Atomic force microscopy of $CuScP_2S_6$ flake. b) Exfoliated $CuScP_2S_6$ flake shows a measured thickness of 30 nm with almost negligible surface roughness.*

Supplementary Figure 2

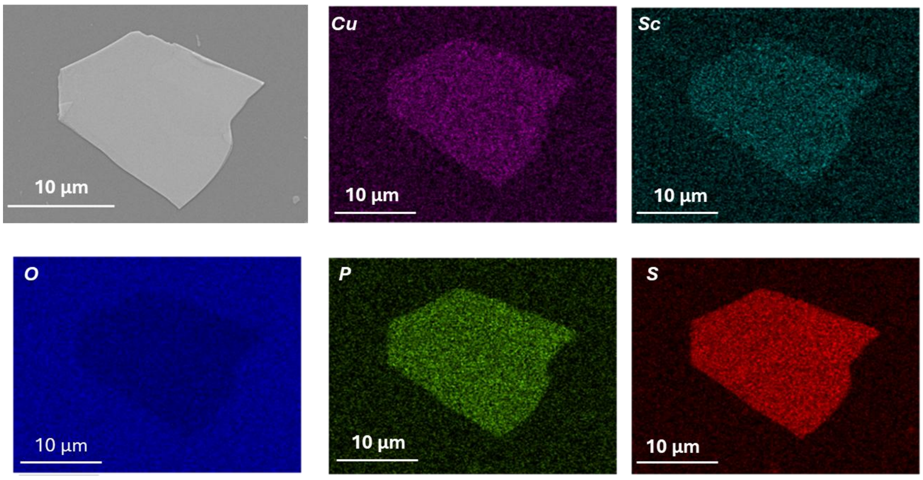

Supplementary Figure 2. SEM and Energy dispersive X-ray spectroscopy (EDS) of an exfoliated $CuScP_2S_6$. *Scanning electron microscopy (SEM) image of $CuScP_2S_6$ flake. EDS verifies the presence of $CuScP_2S_6$ constituents.*

Supplementary Figure 3

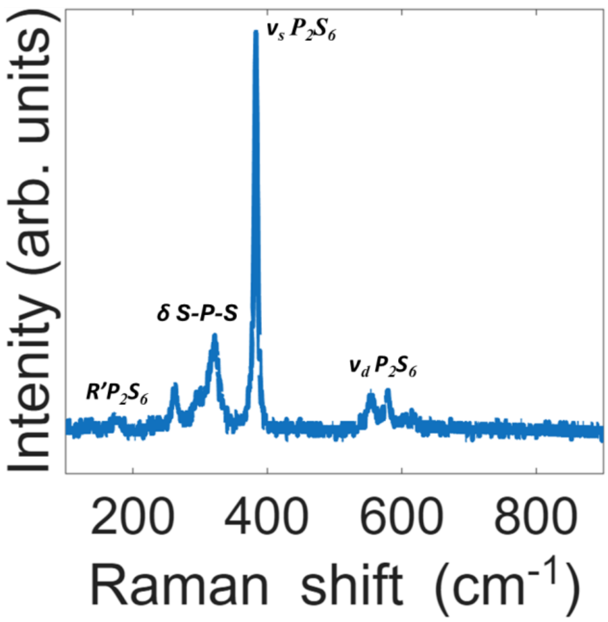

Supplementary Figure 3. Raman Spectroscopy of $CuScP_2S_6$. *Raman spectrum of $CuScP_2S_6$ crystal measured using a 532 nm excitation laser. Raman spectra exhibit four well-resolved vibrational modes associated with the internal dynamics of the $[P_2S_6^-]$ unit, including S-P-P and S-P-S deformations, P-P symmetric stretching, and P-S oscillations. Subtle variations in the P-P stretching mode suggest minor lattice distortions.*

Supplementary Figure 4

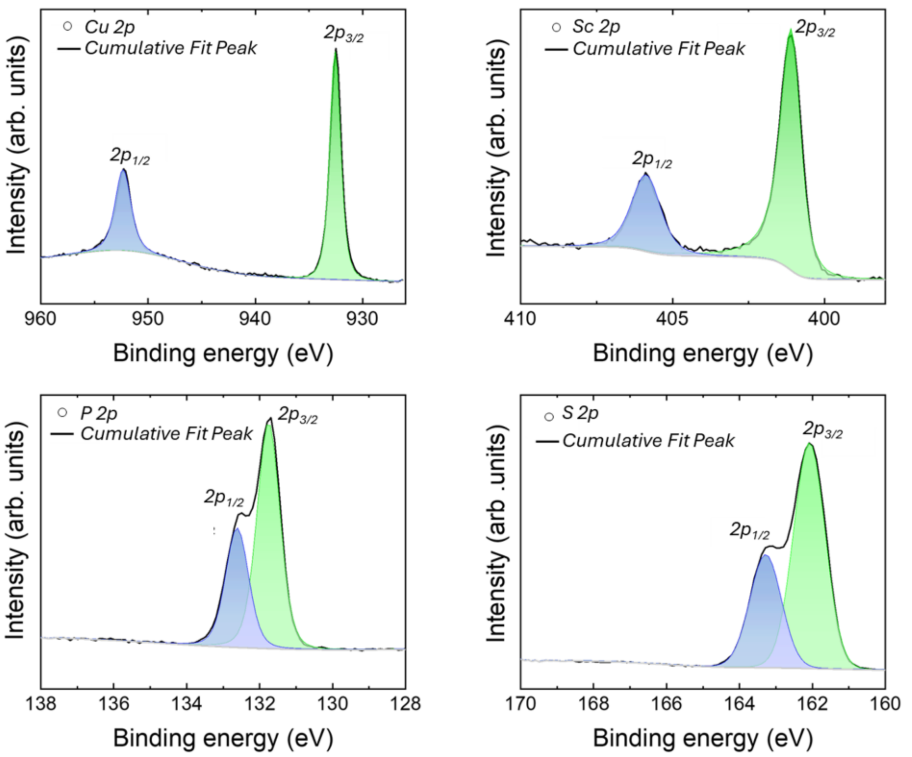

Supplementary Figure 4. X-Ray photoelectron spectroscopy of $CuScP_2S_6$. *X-ray photoelectron spectroscopy (XPS) spectra of a $CuScP_2S_6$ crystal exhibits core level Cu 2p, Sc 2p, P 2p, and S 2p orbitals.*

Supplementary Figure 5

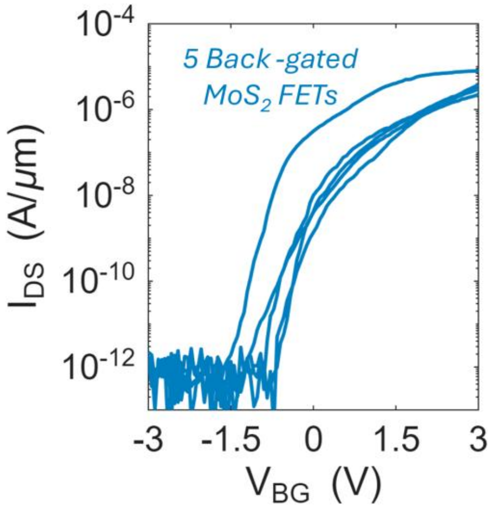

Supplementary Figure 5. Back-gated MoS_2 FETs*. Back-gate transfer characteristics of 5 MoS_2 FETs were assessed by sweeping the V_{BG} from -3 V to 3 V for V_{DS} =1 V.*

Supplementary Figure 6

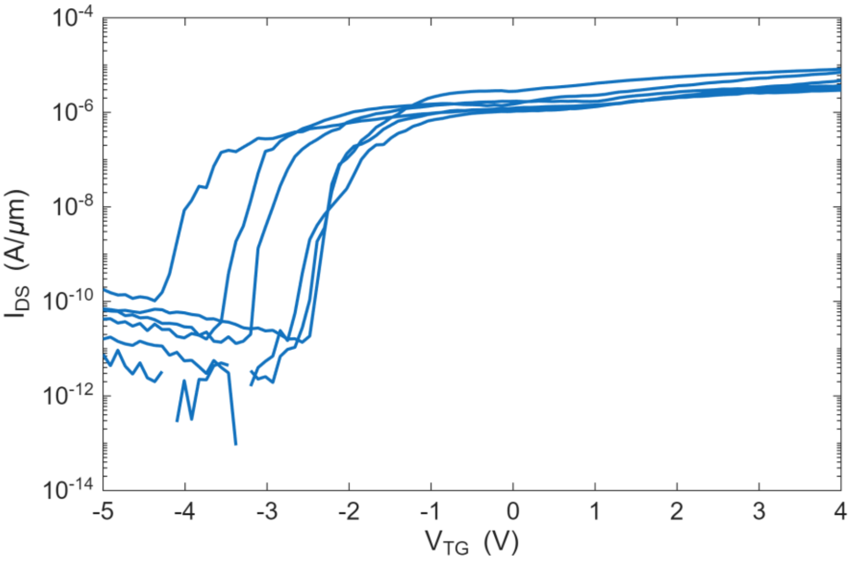

***Supplementary Figure 6. Top-gated transfer characteristics of $CuScP_2S_6$-gated MoS_2 FET**. An ON-current of ~5 µA/µm was obtained for a channel length of 1 µm in 6 $CuScP_2S_6$-gated MoS_2 FET by sweeping the V_{TG} at a constant V_{BG} = 3V and V_{DS} =1 V.*

Supplementary Figure 7

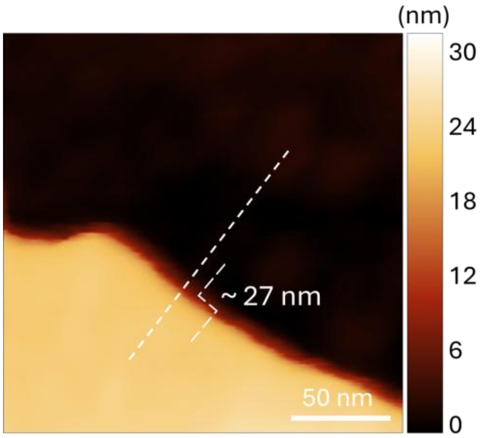

Supplementary Figure 7. Atomic force microscopy (AFM) image of $CuScP_2S_6$ as a top-gate dielectric. *AFM image of an exfoliated $CuScP_2S_6$ flake used as a top-gate dielectric for MoS_2 FET, with a measured thickness of ~27 nm.*

Supplementary Figure 8

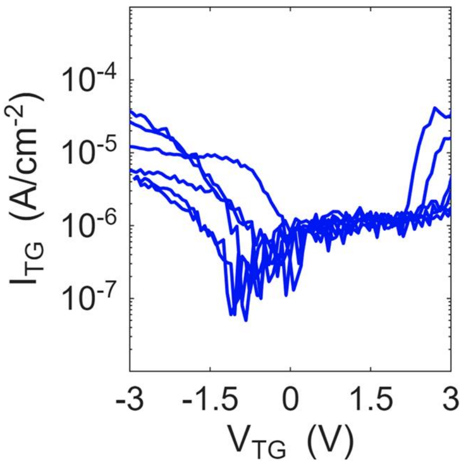

Supplementary Figure 8. Gate leakage current of $CuScP_2S_6$-gated MoS_2 FET. *Gate leakage current of 5 representative MoS_2 FETs gated with $CuScP_2S_6$ were measured by sweeping the V_{TG} at a constant V_{BG} = 3V and V_{DS} =1 V.*

Supplementary Figure 9

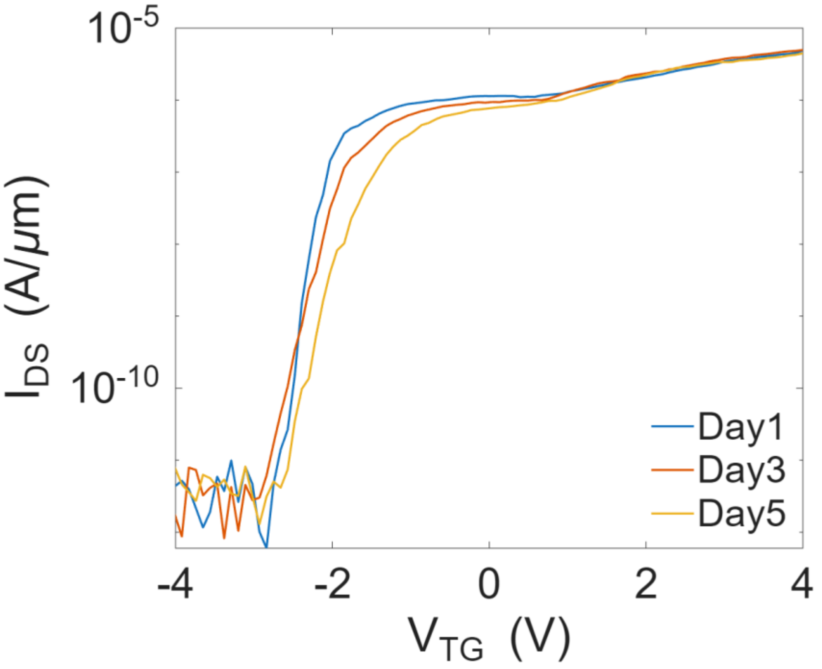

Supplementary Figure 9. Day by day top-gated transfer characteristics of $CuScP_2S_6$ ***-gated*** MoS_2 ***FET****. Day by day top gate characteristics of* $CuScP_2S_6$*-gated* MoS_2 *FET by sweeping the* V_{TG} *at a constant* V_{BG} *= 3V and* V_{DS} *=1 V. There is negligible variation in threshold voltages (*V_{th}*) and no systematic degradation in on/off ratio or subthreshold behaviour over this period, indicating good stability of the* $CuScP_2S_6$ *dielectric and the* $CuScP_2S_6/MoS_2$ *interface.*

Supplementary Figure 10

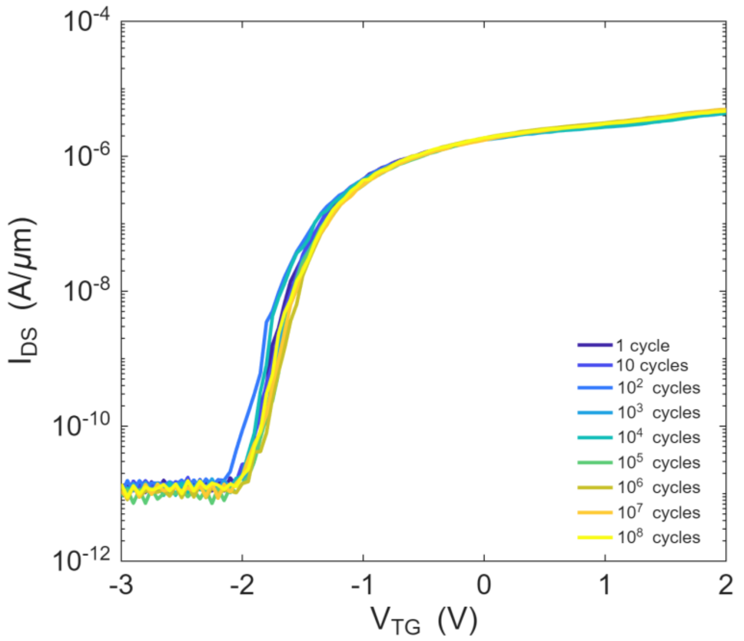

Supplementary Figure 10. Endurance measurement of $CuScP_2S_6$ -gated MoS_2. *The endurance test is performed after giving multiple pulses of 3V at the top gate bias (V_{TG}), ranging from a single pulse to 10^8 pulses. The top gate characteristics of $CuScP_2S_6$-gated MoS_2 were measured by sweeping the V_{TG} from 2 V to -3 V for V_{BG} =3 V and V_{DS} =1 V, after 10^8 cycles, show negligible variation. This confirms the high operational robustness of the $CuScP_2S_6/MoS_2$ system.*

Supplementary Figure 11

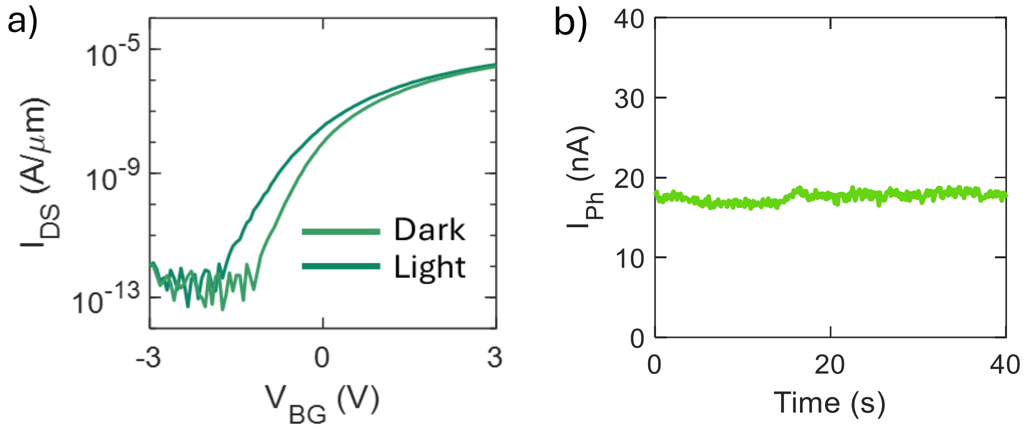

***Supplementary Figure 11. Photoresponse in back gated MoS_2 FET**. a) Back-gate transfer characteristics by sweeping the back-gate voltage (V_{BG}) from under dark and light illumination. b) Time-resolved photoresponse (I_{DS} vs. time) of a representative back-gated MoS_2 FET displaying negligible photoresponse under identical illumination.*

Supplementary Figure 12

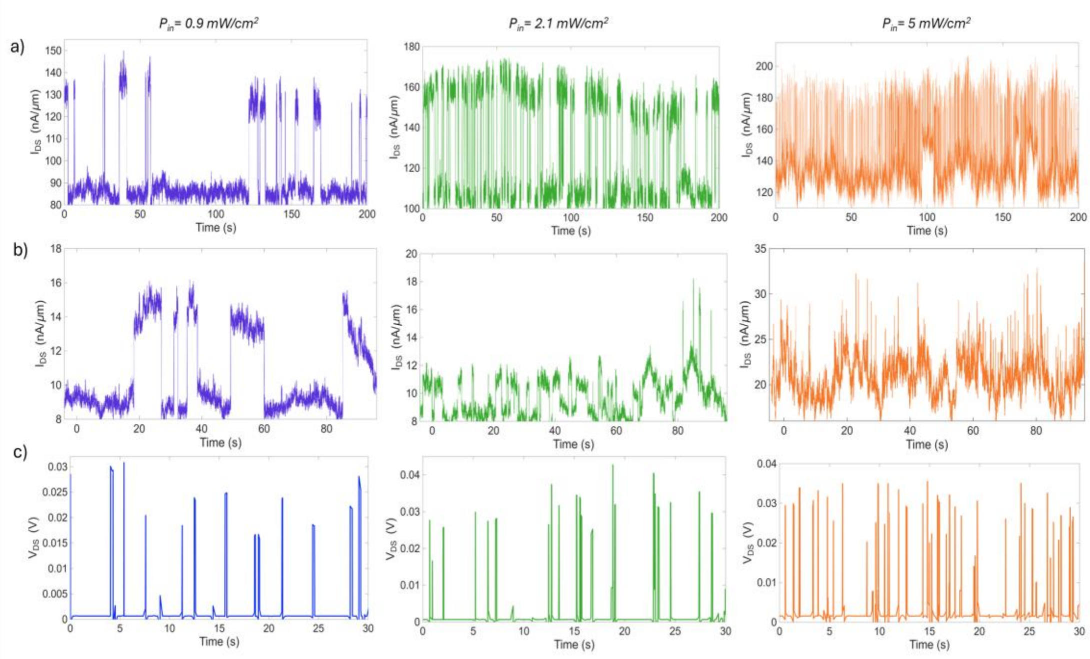

Supplementary Figure 12. Light-induced Random Telegraph noise (RTN) in different $CuScP_2S_6$-gated MoS_2 FETs*. Time-resolved photoresponse shows RTN in different $CuScP_2S_6$-gated MoS_2 FETs for different illumination intensities (P_{in}). a) and b) show time-resolved photoresponse showcasing the light-induced RTN in current (I_{DS}) with different illumination intensity in the representative $CuScP_2S_6$-gated MoS_2 FETs at 200K and 125K, respectively. c) RTN is observed in voltage (V_{DS}) as the time-resolved photo response is measured in a representative device used for stochastic spike encoding for noise-resilient neuromorphic inference at 15K.*

Supplementary Figure 13

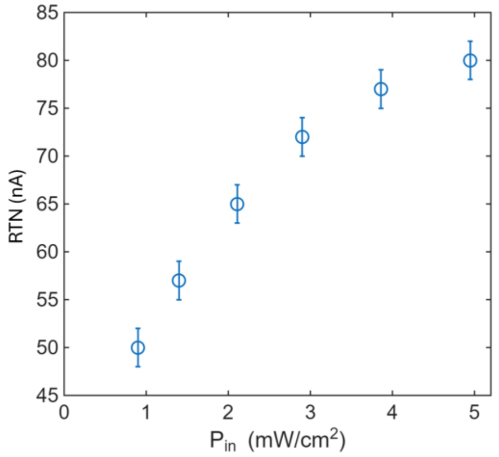

Supplementary Figure 13. Effect of illumination on giant RTN*. RTN amplitude increases from 50 nA at lower illumination (P_{in} = 0.9 mW/cm^2) to 80 nA at higher illumination (P_{in} = 6 mW/cm^2).*

Supplementary Figure 14

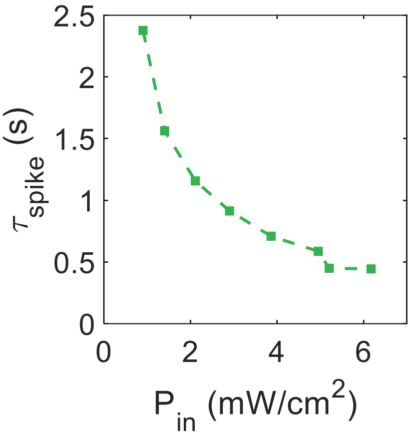

***Supplementary Figure 14. Illumination-dependent spike timing of RTN dynamics in $CuScP_2S_6$-gated MoS_2 FET**. Illumination-dependent evolution of τ_{spike} demonstrates that higher photon flux accelerates the appearance of RTNs.*
