# Supplementary material for: Light-induced giant random telegraph noise in CuScP2S6/MoS2 heterostructures and their use in noise resilience image inference
Source: Nat Commun. 2026 Apr 16;17:5297. doi: 10.1038/s41467-026-71034-6 (PMC13270004; doi:10.1038/s41467-026-71034-6)
Supplement: Supplementary file 2 — Transparent Peer Review File [file 41467_2026_71034_MOESM2_ESM.pdf]

nature portfolio

Peer Review File

Light-induced giant random telegraph noise in CuScP2S6/MoS2 heterostructures and their use in noise resilience image inference

Corresponding Author: Professor Saptarshi Das

This file contains all reviewer reports in order by version, followed by all author rebuttals in order by version.

Version 0:

Reviewer comments:

Reviewer #1

(Remarks to the Author)
The presented results are very original and scientifically very interesting. Light-induced effects on low-frequency noise has been already observed and studied but not in the case of random telegraphic noise (RTN) to the best of my knowledge. The giant light-induced RTN is therefore a very original result and even more in the case of mesoscopic device geometries. The scientific approach is very grumpy and well detailed. The interpretations of the results are overall very convincing.
The section on the control of RTN kinetics and spike statistics through illumination strength is very well presented and convincing. A discussion on the nature of photoactive traps and their origin would certainly be useful. Are these defects intrinsic to the 2D materials used or are they related to $CuScP_2S_6$ or other materials or even the manufacturing process?
The last part of the paper presenting the application to noise tolerant neuromorphic encoding is also original from my point of view.

Reviewer #2

(Remarks to the Author)
In this manuscript, the authors reported the fabrication of a high-k/MoS2 hetero-FET and characterisation of the device under the light illumination to explore the random-telegraph-signals at low temperatures. They also applied the device to use for neural networks. The experimental data is extensive from material characterisations including AFM, SEM, EDS, and Raman, electric measurements including cryogenic dependence, and photo-induced memory effects, to applications for AIs. In particular, the RTN signals under illumination at low temperatures are systematically investigated. I believe this work is a valuable contribution for the material science and device physics of a 2D material. However, considering the potential impact deemed for a publication in Nature Communications, I must give critical comments to clarify the scope of the work. Here, I will feedback to authors my major concerns:

(1) Lack of strong motivation to justify the warranty to publish in the high impact journal:
It might be true that the photon-induced RTN is not explored in this gate stack for the MoS2. However, the NV centres in diamonds, just for example, are very famous in defect engineering for various quantum technologies. I do not see such a great breakthrough, unfortunately, from the present work. It is very important to convince readers why this particular combination of materials is innovative. At least, the introduction and the abstract of the current version is not strong enough.

(2) Application to a neural network is not convincing:
Can you really apply this device for neural networks, given the fact that the device must be operated for tens of seconds under low temperatures with illuminations? It must be true that the authors could use the noise somehow for neural networks. But, considering the neural networks are current hot topics for practical applications, it is far beyond to insist the current device, exceeding beyond the existing frameworks. I am sorry for the critical comments but considering the current device performance and the existing state-of-the-art, I think it is not convincing to discuss the practical applications. In my view, it is better not to include Fig. 5 and related discussions at least in the main text.

(3) Lack of evidence that the trapping/de-trapping realised in the high-k layer:
The RTN signals and 1/f characteristics were successfully observed in the proposed device, but there is no clear evidence

that the memory effects are realised due to the high-k layer. Do they explore the control sample without the high-k layer, which does not show the memory effects? How many devices were examined to confirm the reproducibility? Is this RTN coming from a single trap or multiple traps? Why back gate characteristics shown in Extended Fig.5 are not stable, while the authors obtained the systematic top-gate characteristics in Fig. 4 at 200K?

(4) Here is the list of minor issues:
p.2: light-induced RTN is rarely observed: Is this true? I believe there are a few works for implanted NV centres even without RTN.
p.3, 4: vdW, MNIST, CVT: please spell out
p.14: defect kinetics: is there any evidence that the defect is moving?
p.14: NRMP: please cite a paper.
p.16: most existing encoders…sensors: please cite a paper.

In conclusion, I cannot recommend the publication in Nature Communications, unfortunately. It is recommended to explore elsewhere focussing on material and device characterisation.

Reviewer #3

(Remarks to the Author)
The manuscript by Ghosh et al. reports on CuScP2S6/MoS2-based devices, used as optical encoders. The authors strong, light-induced random telegraph noise, employed to convert image pixels into RTN-driven spike trains. It is claimed that specific properties of CuScP2S6 make the material into model system for future works.
The manuscript is well written, and the results are of potential interest for the readership of the journal. There are however some points, needed to be addressed prior to a decision on the manuscript.

Comments
1. Strong RTN signals are observed only at 200 K. What is the physical reason/effect for this result?

2. Is there a chemical and/or physical interaction between CuScP2S6 and MoS2? Are defects at MoS2 playing some role?

3. Devices are prepared and tested, exposed as well to atmosphere. Is there some effect of the environment (e.g. adsorbed or absorbed moisture, O2 or CO2)?More over in preparation steps H2S is used, which is a source not inly od S, but as well of protons. Such effects can also influence the RTN and devices behaviour in general.

4. What is the stability of CuScP2S6 with time? Are there some ageing effects.

5. I am missing some more general information on the devices: how many devices were tested and what is the device-to-device and sample-to-sample variation? I also see endurance of 100 cycles (ext. data Fig. 8) which is not much… How reliable is the system in general?

6. I am also missing the performance of the devices in comparison to similar devices and systems.

Version 1:

Reviewer comments:

Reviewer #2

(Remarks to the Author)
I have examined all the revisions and comments from the authors and reviewers. I believe this is a minor revision and unfortunately I cannot change my opinion that the paper is suitable for the high impact journal publication, while it will be certainly publishable in a suitable journal.

Specifically, the authors claim there is no serious attempt to utilise the RTN at all, which is not the case. The RTN signals are routinly used to measure the temperature of a sensor singleelectron-transisotr for silicon based spin qubits. It is also well known to be used as an Elzerman readout for high fidelity senseing applications. It is recommended to modify the introduction and the conclusion, accordingly.

Reviewer #3

(Remarks to the Author)
The authors have addressed my comments and imporved the text. I still think that impurties comming especially from H2S (but as well H2O, CO2) are possible to be incorporated into the material and impact the film, but this modifications would give a systematic shift, thus not affecting the conclusion derived.

In my opinion the manuscript can be accepted for publication

Open Access This Peer Review File is licensed under a Creative Commons Attribution 4.0 International License, which permits use, sharing, adaptation, distribution and reproduction in any medium or format, as long as you give appropriate credit to the original author(s) and the source, provide a link to the Creative Commons license, and indicate if changes were made.
In cases where reviewers are anonymous, credit should be given to 'Anonymous Referee' and the source.
The images or other third party material in this Peer Review File are included in the article's Creative Commons license, unless indicated otherwise in a credit line to the material. If material is not included in the article's Creative Commons license and your intended use is not permitted by statutory regulation or exceeds the permitted use, you will need to obtain permission directly from the copyright holder.
To view a copy of this license, visit https://creativecommons.org/licenses/by/4.0/

Reviewer #1 (Remarks to the Author):
The presented results are very original and scientifically very interesting. Light-induced effects on low-frequency noise has been already observed and studied but not in the case of random telegraphic noise (RTN) to the best of my knowledge. The giant light-induced RTN is therefore a very original result and even more in the case of mesoscopic device geometries. The scientific approach is very grumpy and well detailed. The interpretations of the results are overall very convincing.
The section on the control of RTN kinetics and spike statistics through illumination strength is very well presented and convincing. A discussion on the nature of photoactive traps and their origin would certainly be useful. Are these defects intrinsic to the 2D materials used or are they related to $CuScP_2S_6$ or other materials or even the manufacturing process? The last part of the paper presenting the application to noise tolerant neuromorphic encoding is also original from my point of view.

Thank you for this very positive and thoughtful assessment of our work. We sincerely appreciate the reviewer's recognition of the originality of the giant light-induced RTN, particularly in mesoscopic device geometries, as well as the clarity and rigor of the experimental approach and data interpretation.
We are especially grateful for the reviewer's encouraging comments on the section detailing the illumination-controlled RTN kinetics and spike dynamics, and on the originality of the noise-tolerant neuromorphic encoding concept. These points strongly align with the central motivation of our study.
Regarding the reviewer's important suggestion to further discuss the nature and origin of the photoactive traps, we agree that this is a valuable addition and have expanded the discussion accordingly in the revised manuscript. In brief, our experimental evidence indicates that the dominant photoactive traps are not intrinsic to the MoS_2 channel but instead originate primarily from defect states within the $CuScP_2S_6$ (CSPS) dielectric. This conclusion is supported by (i) the absence of RTN in dark conditions, (ii) the strong correlation between illumination and trap kinetics, (iii) the sub-bandgap photoluminescence observed in CSPS, indicative of optically active defect states, and (iv) the large electrostatic leverage required to produce the observed current step heights in a mesoscopic channel.
In the revised manuscript, we now explicitly discuss plausible microscopic origins of the photoactive traps, attributing them to intrinsic defect species within the CSPS dielectric, such as chalcogen vacancies, cation disorder, and defect complexes, which are known to introduce deep, optically active states in layered thiophosphate systems.
We also clarify the role of fabrication-related disorder. While standard lithographic processes may introduce polymer residues or interfacial contamination, such extrinsic effects typically manifest as weak, broadband 1/f noise or static threshold-voltage shifts, rather than the large-amplitude, two-level RTN that is selectively activated by illumination and confined to a narrow temperature window, as observed here. Importantly, polymer residues or surface adsorbates are not expected to generate light-triggered bistable trap dynamics with current steps on the order of tens of nanoamperes in mesoscopic devices.
We thank the reviewer again for their insightful comments, which have helped us strengthen the physical discussion and further clarify the materials origin of the photoactive traps, thereby improving the overall quality and impact of the manuscript.

Reviewer #2 (Remarks to the Author):

In this manuscript, the authors reported the fabrication of a high-k/MoS2 hetero-FET and characterisation of the device under the light illumination to explore the random-telegraph-signals at low temperatures. They also applied the device to use for neural networks. The experimental data is extensive from material characterisations including AFM, SEM, EDS, and Raman, electric measurements including cryogenic dependence, and photo-induced memory effects, to applications for AIs. In particular, the RTN signals under illumination at low temperatures are systematically investigated. I believe this work is a valuable contribution for the material science and device physics of a 2D material. However, considering the potential impact deemed for a publication in Nature Communications, I must give critical comments to clarify the scope of the work. Here, I will feedback to authors my major concerns:

We thank the reviewer for their careful evaluation of the manuscript and for recognizing the breadth and quality of the experimental work, spanning materials characterization, low-temperature electrical measurements, photo-induced phenomena, and neuromorphic applications. We appreciate the opportunity to clarify the scope and impact of the study in the context of *Nature Communications*.

At its core, this work reports a previously unexplored physical regime of noise in 2D heterostructure transistors: namely, the emergence of giant, illumination-induced random telegraph noise (RTN) that is absent in the dark, selectively activated within a narrow temperature window, and observable in mesoscopic device geometries. While light-induced low-frequency noise and photo-response in 2D devices have been widely studied, RTN itself has almost exclusively been associated with nanoscale confinement, extreme electrostatic scaling, or stochastic defect occupation under purely electrical stress [1, 2]. Demonstrating light-triggered, controllable RTN in a large-area device therefore represents a fundamentally new noise modality, extending the physics of RTN beyond its traditional limits.

Importantly, the manuscript goes beyond reporting an isolated observation. Through systematic temperature-, gate-bias-, and illumination-dependent studies, we establish a consistent physical picture in which photoactive deep traps primarily associated with the high-κ $CuScP_2S_6$ dielectric govern the stochastic switching dynamics. The ability to continuously tune RTN kinetics and spike dynamics using optical power provides an unprecedented level of control over stochastic charge dynamics in 2D heterostructures. This degree of tunability, combined with the large RTN amplitude observed in mesoscopic channels, has not been reported previously and constitutes a key advance in both device physics and defect engineering.

The final part of the manuscript leverages this physical insight to demonstrate a conceptual shift in how noise is treated in electronic systems. Rather than suppressing RTN as a reliability concern, we show that intrinsic, light-controlled stochasticity can be directly harnessed for noise-tolerant neuromorphic encoding, yielding enhanced robustness against input noise compared to deterministic encoding schemes. This connection between defect-driven charge dynamics, optical control, and information processing elevates the work beyond a materials or device study alone, placing it at the intersection of materials science, nanoelectronics, and emerging computing paradigms.

We therefore believe that the manuscript aligns well with the scope of *Nature Communications*, as it (i) reveals a new physical phenomenon, (ii) provides mechanistic understanding supported by extensive experimental evidence, and (iii) demonstrates broad relevance by translating fundamental noise physics into a functional, system-level concept. We hope this clarification addresses the reviewer’s concerns and helps contextualize the significance of the reported results.

1. Lack of strong motivation to justify the warranty to publish in the high impact journal: It might be true that the photon-induced RTN is not explored in this gate stack for the MoS2. However, the NV centres in diamonds, just for example, are very famous in defect engineering for various quantum technologies. I do not see such a great breakthrough, unfortunately, from the present work. It is very important to convince readers why this particular combination of materials is innovative. At least, the introduction and the abstract of the current version is not strong enough.

We thank the reviewer for raising this important point regarding the broader motivation and perceived impact of the work. We agree that the context and framing of the manuscript, particularly in the Abstract and Introduction are critical, and we have revised these sections to more clearly articulate the novelty and significance of our results.

We emphasize that the scientific advance reported here is fundamentally distinct from defect platforms such as NV centres in diamond. NV centres are intentionally isolated, atomically defined quantum defects engineered for spin coherence and quantum sensing, typically operating in highly controlled, optically addressed solid-state environments [3-5]. In contrast, random telegraph noise (RTN) in field-effect transistors represents stochastic charge-state fluctuations of electrically active defects that directly modulate transport in a semiconductor channel [6-8]. RTN is therefore not a spectroscopic probe of a single defect, but a transport-level manifestation of defect physics that governs device reliability, variability, and ultimately circuit behaviour in scaled electronics.

Despite decades of study, RTN has been almost exclusively treated as an undesirable parasitic effect, particularly in aggressively scaled CMOS devices. The key breakthrough of this work is the demonstration that RTN can be optically activated, controlled, and harnessed in a mesoscopic 2D hetero-FET, without relying on extreme scaling or single-defect isolation. This introduces a new control dimension-light-for tuning stochastic charge dynamics in transistors, which has not been previously demonstrated.

The choice of the $CuScP_2S_6/MoS_2$ gate stack is central to this advance. The high-κ, defect-rich van der Waals dielectric provides (i) strong electrostatic coupling to the channel, enabling giant RTN amplitudes, and (ii) optically active deep traps that allow photon-controlled switching kinetics, a combination that is not accessible in conventional oxide or diamond-based systems. Importantly, this behaviour emerges in large-area devices, making it directly relevant to electronic hardware rather than isolated quantum defects.

From an application perspective, our work demonstrates how controlled RTN can be transformed from a liability into a resource. By mapping light-tuneable RTN into spike statistics, we show a concrete pathway for noise-tolerant neuromorphic encoding, addressing a central challenge in hardware AI. More broadly, the ability to generate and control stochastic dynamics in solid-state transistors is highly relevant for probabilistic computing, hardware random number generation, and hybrid quantum-classical interfaces, where defect-driven fluctuations play a functional role.

We believe these clarifications, together with the strengthened Abstract and Introduction, clearly establish why this material system and the reported phenomenon represent a conceptual advance in defect physics and nanoelectronic functionality, aligning well with the scope and impact expected for *Nature Communications*.

2. Application to a neural network is not convincing:

Can you really apply this device for neural networks, given the fact that the device must be operated for tens of seconds under low temperatures with illuminations? It must be true that the authors could use the noise somehow for neural networks. But, considering the neural networks are current hot topics for practical applications, it is far beyond to insist the current

device, exceeding beyond the existing frameworks. I am sorry for the critical comments but considering the current device performance and the existing state-of-the-art, I think it is not convincing to discuss the practical applications. In my view, it is better not to include Fig. 5 and related discussions at least in the main text.

We thank the reviewer for this thoughtful comment regarding the practical applicability of the demonstrated neuromorphic concept. We fully agree that the present devices, which operate under low-temperature and optical excitation conditions, are not intended as immediately deployable hardware for real-world neural network applications. Our goal in presenting Fig. 5 and the associated discussion is not to claim near-term technological readiness, but rather to demonstrate a proof-of-concept functional use of controlled stochasticity emerging from defect-driven RTN in a transistor platform.

The neuromorphic encoding example is included to illustrate a broader point: that RTN traditionally viewed as a reliability concern can be transformed into a computational resource when its statistics are controllable. This conceptual advance is relevant to emerging directions in stochastic, probabilistic, and neuromorphic computing, where device-level noise is increasingly explored as a feature. We have revised the manuscript to clearly state that the present demonstration serves as a conceptual framework rather than a claim of immediate practical competitiveness with existing neuromorphic hardware.

In response to the reviewer's concern, we have also moderated the language in the main text to avoid overstating practical applicability and have clarified the limitations associated with temperature, illumination, and operational timescales. We believe that including Fig. 5 remains valuable in highlighting the system-level implications of the newly discovered light-controlled RTN phenomenon, while appropriately framing it as an exploratory demonstration that may inform future device and materials development toward scalable platforms.

3. Lack of evidence that the trapping/de-trapping realised in the high-k layer:

We thank the reviewer for this important comment regarding the physical location of the trapping/de-trapping process. While we acknowledge that pinpointing the exact microscopic site of individual traps is inherently challenging in complex heterostructures, multiple independent experimental observations in our work consistently indicate that the dominant trapping/de-trapping events originate within the high-κ $CuScP_2S_6$ (CSPS) gate dielectric rather than in the MoS_2 channel.

First, in conventional transistors, discrete random telegraph noise (RTN) is typically observed only in aggressively scaled devices, where transport is dominated by one or a few defects. In long-channel FETs, the collective contribution of many traps generally averages out, resulting in 1/f noise rather than a Lorentzian fit indicating a clean two-state RTN. The observation of large-amplitude, two-state RTN in our MoS_2 devices therefore represents a highly unusual regime and cannot be readily explained by channel-intrinsic or process-induced disorder. Secondly, the complete absence of RTN in the dark, despite measurable current levels and stable operation, strongly argues against intrinsic channel defects [2] or contact-related traps [9], which typically produce RTN independent of illumination.

Moreover, the RTN amplitude reaches tens of nanoamperes in 2D channels, implying a trap with exceptionally strong electrostatic leverage consistent with a trap located in the gate dielectric capacitively coupled across the full channel, rather than a localized defect in the MoS_2 lattice. Notably, the illumination-controlled acceleration of capture and emission kinetics, together with the preservation of a clean two-state RTN signature over a wide range of gate biases, is characteristic of photoactive deep traps and inconsistent with shallow channel states or interface adsorbates. This interpretation is further supported by the sub-bandgap

photoluminescence observed in CSPS, evidencing optically active defect states capable of mediating photon-assisted charge transitions. Furthermore, the temperature-selective activation window of the RTN and its systematic tunability with optical power are naturally explained by trap energetics and carrier emission barriers within the dielectric, whereas channel- or contact-dominated trapping would not exhibit such sharply defined activation behaviour.

Taken together, the emergence of clean RTN in a 2D device only under illumination and with large amplitude provides strong, self-consistent evidence that a small number of electrostatically dominant, photoactive traps within the high-κ CSPS layer govern the observed trapping/de-trapping dynamics. We have clarified this physical reasoning explicitly in the revised manuscript.

4. The RTN signals and 1/f characteristics were successfully observed in the proposed device, but there is no clear evidence that the memory effects are realised due to the high-k layer. Do they explore the control sample without the high-k layer, which does not show the memory effects? How many devices were examined to confirm the reproducibility? Is this RTN coming from a single trap or multiple traps? Why back gate characteristics shown in Extended Fig.5 are not stable, while the authors obtained the systematic top-gate characteristics in Fig. 4 at 200K?

We thank the reviewer for these thoughtful questions, which allow us to clarify several important points regarding the origin, reproducibility, and interpretation of the observed RTN phenomena.

We would like to clarify that the random telegraph noise (RTN) reported in this work is not a memory effect. Unlike non-volatile or photo-induced memory, the RTN manifests as stochastic, reversible trapping/de-trapping events that do not retain a programmed state once the external stimulus (illumination or bias) is removed. The device returns to its original conductance level after light is switched off, and no persistent conductance state is observed. We have now clarified this distinction explicitly in the revised manuscript to avoid confusion between RTN and memory-related phenomena.

While we do not claim that the device exhibits memory due to the high-κ layer, the RTN itself is strongly linked to the presence of the $CuScP_2S_6$ (CSPS) dielectric. Control MoS_2 devices without the CSPS top-gate dielectric (back-gated MoS_2 FETs fabricated using the same process flow) do not exhibit light-activated, two-level RTN under otherwise identical measurement conditions. This comparison supports the conclusion that the photoactive trapping responsible for RTN is associated with the high-κ CSPS layer, rather than the MoS_2 channel or contacts.

The RTN behaviour reported here was observed consistently across multiple devices fabricated on different flakes as shown in **Fig. R1**. We would also like to clarify that the reported phenomena are not device-specific anomalies. We have clarified the number of tested devices and the reproducibility statistics in the revised manuscript. Notably, the observed RTN is best described as arising from a small number of electrostatically dominant traps, rather than a single isolated defect or a large ensemble of weak traps. This is evidenced by the clean two-state switching, narrow dwell-time distributions, and Lorentzian-like power spectral density under illumination. At certain gate biases and temperatures, one trap dominates the conductance modulation, producing an effective two-state RTN, while at other biases weak multi-level fluctuations can emerge. We have clarified this point to avoid over-interpretation in terms of a strictly single-trap picture.

The reviewer correctly notes that the back-gate transfer characteristics in Extended Data Fig. 5 exhibit larger variability. We clarify that this figure is intended to show representative back-gated operation across multiple devices, establishing baseline transistor functionality and

reproducibility, rather than optimized or systematic electrostatic control. The observed variation is expected for substrate-gated MoS_2 FETs, where transport is sensitive to interface trap charges and fabrication-related residues at the MoS_2/Al_2O_3 interface, leading to device-to-device threshold shifts. In contrast, Fig. 4 focuses on time-domain measurements at fixed bias conditions selected to probe light-induced RTN dynamics, rather than on sweeping transfer characteristics. We have clarified this distinction in the revised manuscript to avoid a direct comparison between the two measurement configurations.
In summary, the revised manuscript clearly distinguishes RTN from memory effects, demonstrates reproducibility across multiple devices, explains the origin of RTN as arising from a small number of dominant traps in the high-κ layer, and clarifies the differing behaviour observed in back-gate versus top-gate measurements. We thank the reviewer again for these constructive comments, which have helped improve the clarity and rigor of the presentation.

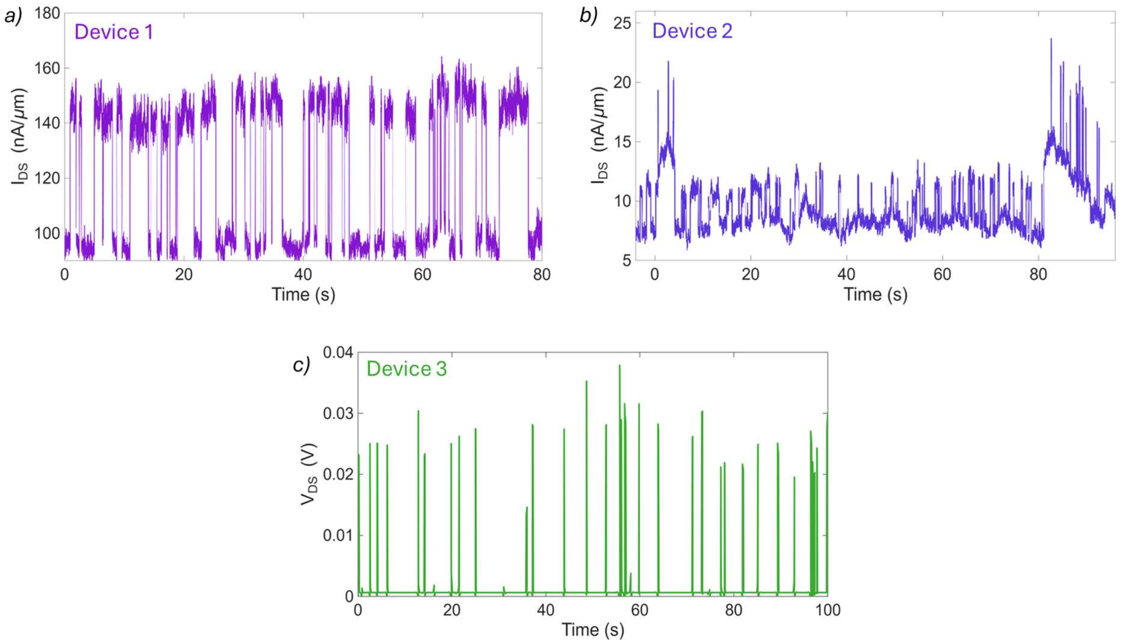

Figure R1: Light-induced Random Telegraph noise (RTN) in different $CuScP_2S_6$-gated MoS_2 FETs. Time-resolved photoresponse shows RTN in different $CuScP_2S_6$-gated MoS_2 FETs . *Time-resolved photoresponse showing RTN in **a), b)** current **(I_{DS})** for two different devices **c)** in voltage **(V_{DS})**. Multiple devices showed the presence of RTN in both the current and voltage domains. Device 3 is being used of the stochastic spike encoding for the noise resilient neuromorphic inference, for which, instead of current, voltage is measured as voltage spikes are naturally suited for neuromorphic encoding and direct interfacing with peripheral circuits.*

(4) Here is the list of minor issues:
p.2: light-induced RTN is rarely observed: Is this true? I believe there are a few works for implanted NV centres even without RTN.
We agree that light–defect interactions have been widely studied in various material systems, including well-known platforms such as NV centres in diamond, and we do not intend to suggest that photoactivated defect dynamics are entirely unexplored. However, light-induced random telegraph noise (RTN) in field-effect transistors is rarely reported, and, to the best of our knowledge, has not been systematically observed and controlled in nanoscale 2D FETs. In NV-centre–based and related quantum-defect systems, optical excitation typically addresses isolated defects for spectroscopic or spin-based functionalities and does not manifest as

stochastic modulation of channel conductance. By contrast, RTN in transistors arises from charge trapping/de-trapping events that directly impact electronic transport and has traditionally been associated with electrical stress or aggressive scaling rather than optical control. We have therefore revised the manuscript to clearly qualify this distinction and emphasize that the central advance of this work is the demonstration of optical activation and tunability of RTN in a transistor platform, which represents a largely unexplored regime of defect–transport physics.

p.3, 4: vdW, MNIST, CVT: please spell out
We have included the suggested changes in the revised manuscript.

p.14: defect kinetics: is there any evidence that the defect is moving?
We thank the reviewer for this question. We would like to clarify that the observed RTN does not require physical motion of defects. The experimental signatures are consistent with reversible charge-state fluctuations of localized defect states, i.e., trapping and de-trapping of carriers, rather than spatial migration of defects or ions. This interpretation is supported by the discrete two-state switching, well-defined dwell-time statistics, and the absence of cumulative drift or irreversible conductance changes during repeated illumination cycles. While we cannot completely exclude subtle atomic rearrangements at the microscopic level, there is no experimental evidence suggesting long-range defect motion, and the data are most naturally explained by electronic defect kinetics. We have clarified this distinction in the revised manuscript.

p.14: NRMP: please cite a paper.
We have cited relevant papers [10, 11] in the revised manuscript.

p.16: most existing encoders…sensors: please cite a paper.
We have cited relevant papers [12, 13] in the revised manuscript.

In conclusion, I cannot recommend the publication in Nature Communications, unfortunately. It is recommended to explore elsewhere focussing on material and device characterisation.

We thank the reviewer for the careful evaluation. While we respectfully disagree with the conclusion, we emphasize that this work reports a new transport-level regime in which random telegraph noise (RTN) is optically activated and controllable in a 2D transistor, rather than presenting incremental material or device characterization. The results demonstrate that RTN that has been traditionally linked to aggressive scaling and electrical stress can instead be accessed and tuned using light, offering new insight into defect-driven charge dynamics and enabling functional stochastic encoding for neuromorphic systems. We believe this conceptual advance and its broad relevance align well with the scope of *Nature Communications*, and the manuscript has been revised to better convey this significance.

Reviewer #3 (Remarks to the Author):
The manuscript by Ghosh et al. reports on $CuScP_2S_6/MoS_2$-based devices, used as optical encoders. The authors strong, light-induced random telegraph noise, employed to convert image pixels into RTN-driven spike trains. It is claimed that specific properties of $CuScP_2S_6$ make the material into model system for future works. The manuscript is well written, and the results are of potential interest for the readership of the journal. There are however some points, needed to be addressed prior to a decision on the manuscript.

We thank the reviewer for their positive and constructive assessment of the manuscript. We appreciate their recognition that the $CuScP_2S_6/MoS_2$ heterostructures demonstrate strong light-induced RTN that can be harnessed for optical encoding, and that the results are of potential interest to the journal's readership. We also thank the reviewer for acknowledging the clarity of the presentation and the promise of $CuScP_2S_6$ as a model vdW dielectric platform for future studies. We welcome the reviewer's specific points and have addressed each of the concerns raised in detail below, with corresponding clarifications and revisions incorporated into the manuscript to strengthen the physical interpretation, experimental justification, and broader relevance of the work.

Comments
1. Strong RTN signals are observed only at 200 K. What is the physical reason/effect for this result?

We thank the reviewer for the question regarding the temperature dependence of the random telegraph noise (RTN). We clarify that light-induced RTN is observed over a broad temperature range, including 15, 100, and 200 K, demonstrating that the underlying photoactive defect states are not restricted to a narrow temperature window (**Fig. R2**). Nevertheless, the RTN exhibits a non-monotonic temperature dependence that is consistent with a thermally activated trapping–detrapping mechanism. At higher temperatures (~250 K), the trap kinetics become too fast relative to the measurement bandwidth, causing the fluctuations to average out in time. At lower temperatures (≤ 150 K), the traps are effectively frozen into a single charge configuration over the measurement window, suppressing resolvable switching events. In contrast, at an intermediate temperature of ~200 K, the characteristic trapping and detrapping times become comparable to the integration time, resulting in clearly resolvable two-level fluctuations. Notably, the RTN amplitude in this regime is exceptionally large, with discrete current steps of approximately 60 nA. This giant RTN indicates that photoactivated charge rearrangements within the CSPS dielectric induce substantial, quantized shifts in the effective top-gate potential, thereby strongly modulating the MoS_2 channel conductance.
The main quantitative analysis therefore focuses on measurements at 200 K, where the RTN displays the most stable two-state behaviour, with reproducible amplitudes and well-defined dwell-time statistics. This enables reliable extraction of switching kinetics and spike statistics. More generally, low-temperature measurements are essential for RTN analysis, as reduced thermal noise and suppressed competing transport fluctuations allow discrete trapping and detrapping events to be resolved in the time domain. Although RTN is evident at other temperatures, the fluctuations are less stable over extended measurement periods, rendering systematic statistical analysis less reliable. These clarifications have been incorporated into the revised manuscript, where we explicitly state that RTN persists across multiple temperatures, with 200 K identified as the optimal regime for detailed quantitative evaluation.

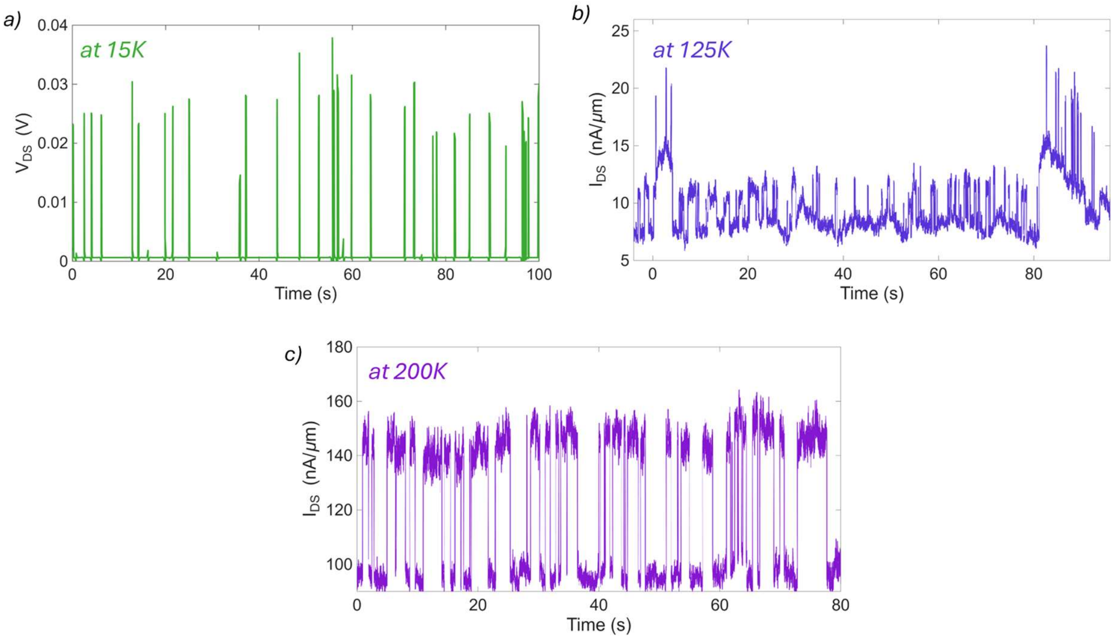

Figure R2: Light-induced Random Telegraph noise (RTN) in $CuScP_2S_6$ -gated MoS_2 FETs at different temperatures. *Time-resolved photoresponse shows RTN in different CuScP2S6-gated MoS2 FETs at different temperatures:* ***a)*** *15K*, ***b)*** *125K*, ***c)*** *200K*. *Based on the flack thickness and active trap states available for different temperatures, different devices show RTN both in current and voltage domain. The representative device which worked at 15K was used for the stochastic spike encoding for the noise resilient neuromorphic inference, for which, instead of current, voltage is measured as voltage spikes are naturally suited for neuromorphic encoding.*

2. Is there a chemical and/or physical interaction between CuScP2S6 and MoS2? Are defects at MoS2 playing some role?

We thank the reviewer for this important question regarding possible chemical and/or physical interactions between $CuScP_2S_6$ (CSPS) and MoS_2, and the potential role of MoS_2 defects. The CSPS/ MoS_2 heterostructure is assembled via van der Waals stacking without intentional chemical functionalization. While our structural and spectroscopic characterizations do not show evidence of macroscopic interfacial reactions or compound formation, we note that subtle interfacial interactions or defect formation at the atomic scale cannot be entirely excluded.
Regarding the origin of the observed RTN, while we cannot completely exclude a secondary contribution from interfacial states or intrinsic defects in MoS_2, multiple experimental observations indicate that the dominant photoactive traps responsible for the light-induced RTN are not primarily located in the MoS_2 channel. In particular, the absence of RTN in the dark, the strong illumination-dependent tunability of the switching kinetics, the large RTN amplitude in long-channel 2D FETs, and the presence of optically active defect states in CSPS (evidenced by sub-bandgap photoluminescence) collectively point to deep, photoactive defect states associated with the CSPS dielectric as the main contributors.
We therefore interpret the CSPS layer as providing a defect-rich, optically active electrostatic environment that enables light-controlled charge trapping/de-trapping, which is then transduced into conductance fluctuations in the MoS_2 channel. While MoS_2 defects and interface states may influence the baseline transport and threshold voltage, the data support the

conclusion that the light-induced RTN is governed primarily by defect states in the CSPS dielectric rather than by chemical interaction or defect dynamics intrinsic to MoS_2.

3. Devices are prepared and tested, exposed as well to atmosphere. Is there some effect of the environment (e.g. adsorbed or absorbed moisture, O2 or CO2)? More over in preparation steps H2S is used, which is a source not only of S, but as well of protons. Such effects can also influence the RTN and devices behaviour in general.

We thank the reviewer for raising this important point regarding possible environmental influences (e.g., adsorbed moisture, O_2, CO_2) and the potential impact of H_2S exposure during preparation. We note that while the devices are fabricated under ambient conditions during certain steps, all RTN measurements reported in this work are performed under high vacuum and at low temperatures, which strongly suppress the presence and mobility of physiosorbed species. Under these measurement conditions, adsorbed moisture is expected to be largely desorbed or immobilized, minimizing the contribution to dynamic trapping/de-trapping processes.

Regarding the use of H_2S during preparation, we acknowledge that H_2S can act as a sulphur source and, in principle, may introduce hydrogen-related species. However, the H_2S treatment is applied during material synthesis rather than device operation, and no signatures of protonic or ionic migration (e.g., slow drift, cumulative hysteresis, or irreversible conductance changes) are observed in our low-temperature, high-vacuum measurements. The RTN observed here is reversible, stochastic, and illumination-controlled, which is inconsistent with slow environmental adsorption/desorption or proton migration processes that typically manifest as long-timescale drift or bias-stress effects.

Moreover, the absence of RTN in the dark and its reproducible activation only under illumination further argue against an environmental origin, as adsorbates and residual species would be expected to influence transport even without optical excitation. While we cannot completely exclude a static influence of residual adsorbates on baseline device characteristics, the data support the conclusion that the observed light-induced RTN is governed by intrinsic photoactive defect states in the CSPS dielectric, rather than by dynamic environmental effects.

4. What is the stability of CuScP2S6 with time? Are there some ageing effects.

We thank the reviewer for this important question regarding the time stability and potential ageing effects of $CuScP_2S_6$ (CSPS). To directly address this concern, we have evaluated the temporal stability of the CSPS-gated devices by repeatedly measuring the top-gate transfer characteristics of a representative CSPS-gated MoS_2 FET over five consecutive days under identical measurement conditions as shown in **Fig. R3**. We observe negligible variation in the threshold voltage (V_{th}) and no systematic

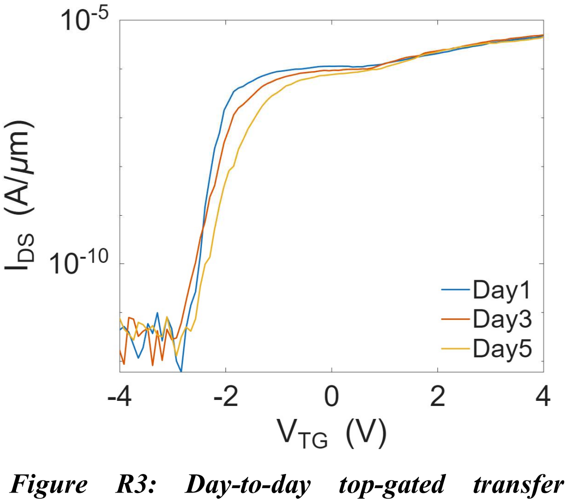

Figure R3: Day-to-day top-gated transfer characteristics of $CuScP_2S_6$ -gated MoS2 FET*.* *Day to day variation in the top gate transfer characteristics of $CuScP_2S_6$ -gated MoS_2 FET by sweeping the V_{TG} at a constant V_{BG} = 3V and V_{DS} =1 V. There is minimal variation, indicating good stability of the $CuScP_2S_6$ dielectric and the $CuScP_2S_6$/MoS_2 interface.*

degradation in on/off ratio or subthreshold behaviour over this period, indicating good stability of the CSPS dielectric and the CSPS/MoS_2 interface. These repeated measurements demonstrate that the CSPS-gated devices exhibit stable electrical characteristics over multiple days, confirming that the observed RTN is a robust and reproducible phenomenon rather than a transient artefact of device instability.

5. I am missing some more general information on the devices: how many devices were tested and what is the device-to-device and sample-to-sample variation? I also see endurance of 100 cycles (ext. data Fig. 8) which is not much… How reliable is the system in general?

We thank the reviewer for this important question regarding device statistics, variability, and reliability. Notably, we have tested multiple CSPS-gated MoS_2 FETs with different flake thicknesses, and the key observations, namely the emergence of light-induced RTN, its illumination-dependent tunability, and reproducible stochastic spike generation—are consistently observed across representative devices (**Fig. R4**). While some degree of device-to-device and sample-to-sample variation is expected in 2D heterostructure devices due to differences in flake thickness, interface quality, and contact resistance, the qualitative RTN behaviour and its controllability remain robust. We have clarified the number of devices examined and the representative nature of the data shown in the revised manuscript.
Regarding endurance, we note that the 100-cycle endurance shown in Extended Data Fig. 8 is not intended to represent the intrinsic reliability limit of the system, but rather a proof-of-concept demonstration of reversible operation under repeated optical/electrical cycling.

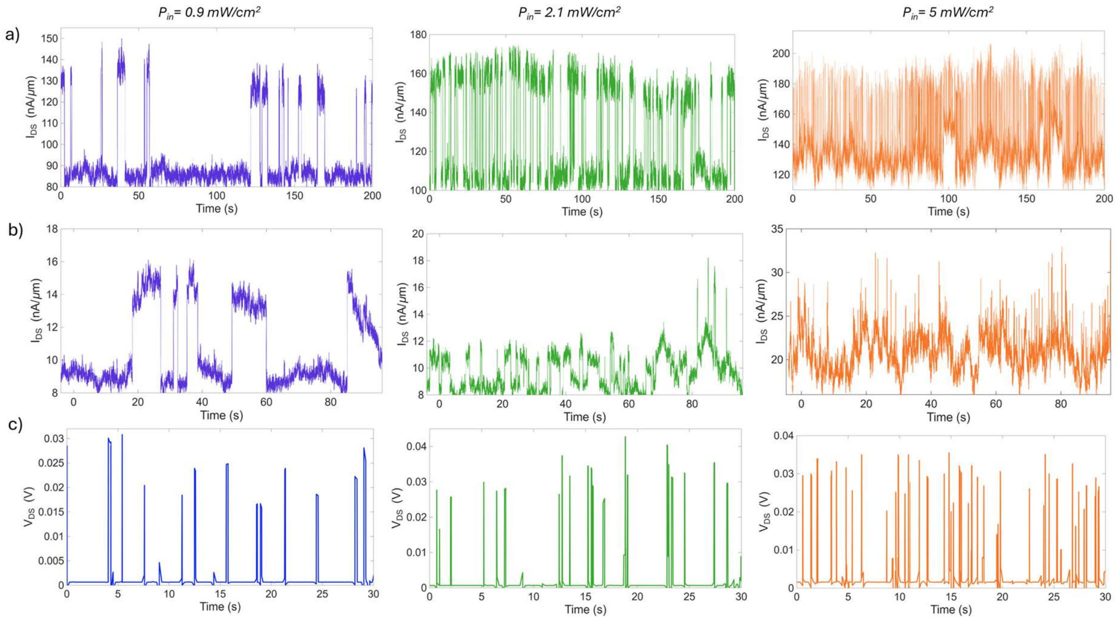

Figure R4: Light-induced Random Telegraph noise (RTN) in different $CuScP_2S_6$-gated MoS_2 FETs. *Time-resolved photoresponse shows RTN in different $CuScP_2S_6$-gated MoS_2 FETs for different illumination intensities* ***(P_{in}). a)*** *and* ***b)*** *show time-resolved photoresponse showcasing the light-induced RTN in current (I_{DS}) with different illumination intensity in the representative $CuScP_2S_6$-gated MoS_2 FETs.* ***c)*** *RTN is observed in voltage* ***(V_{DS})*** *as the time-resolved photoreseponse is measured in a representative device used for stochastic spike encoding for noise-resilient neuromorphic inference.*

Importantly, we have performed extensive endurance tests exceeding up to 10^8 cycles exhibiting negligible variations in top-gate transfer characteristics as shown in **Fig. R5**. These additional endurance measurements confirm that the system exhibits high operational robustness. We have updated the manuscript to clarify this point and better reflect the overall reliability of the platform.

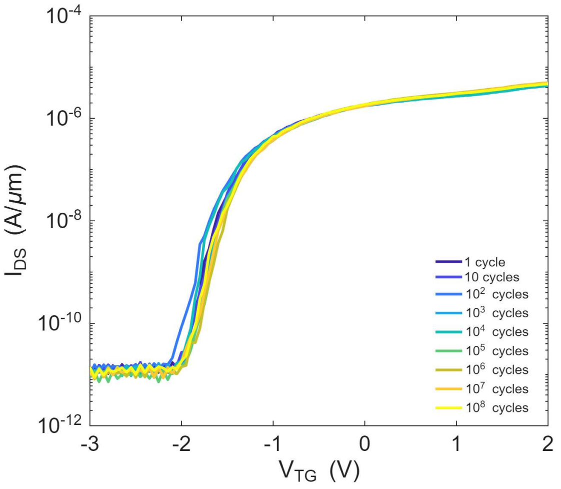

Figure R5: Endurance measurement of $CuScP_2S_6$-gated MoS_2. *The endurance test is performed after giving multiple pulses of 3V at the top gate bias (V_{TG}), ranging from a single pulse to 10^8 pulses. The top gate characteristics of $CuScP_2S_6$-gated MoS_2, measured by sweeping the V_{TG} from 2 V to -3 V for V_{BG} =3 V and V_{DS} =1 V, after 10^8 cycles, show negligible variation. This confirms the high operational robustness of the $CuScP_2S_6/MoS_2$ system.*

6. I am also missing the performance of the devices in comparison to similar devices and systems.

We thank the reviewer for this suggestion regarding performance comparison with related devices and systems. We agree that contextualizing the device behaviour within the broader

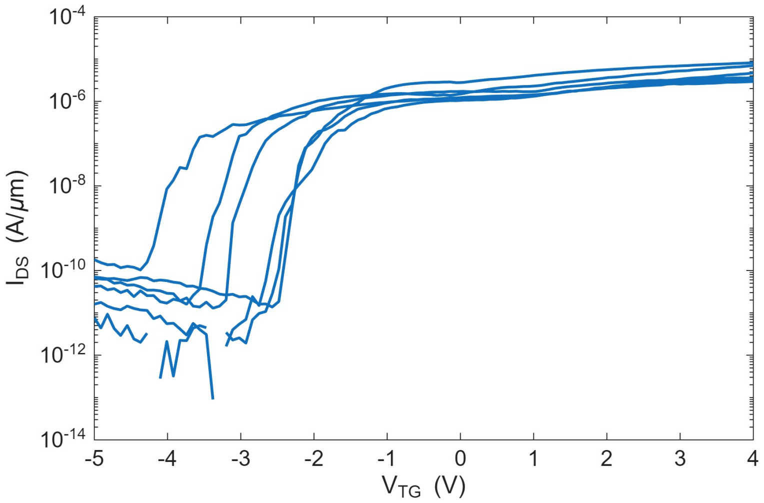

Figure R6. Top-gated transfer characteristics of $CuScP_2S_6$-gated MoS2 FET. *~10 μA/μm for a channel length of 1 μm were measured in 6 $CuScP_2S_6$-gated MoS2 FET by sweeping the V_{TG} at a constant V_{BG} = 3V and V_{DS} =1 V.*

landscape is important. We note that the primary contribution of this work is not the optimization of conventional transistor performance metrics (e.g., on-state current, on/off ratio, or subthreshold swing), but rather the demonstration of a new functional regime such as light-controlled RTN in a 2D hetero-FET and its use for stochastic optical encoding.

That said, the electrical performance of our CSPS-gated MoS_2 devices (on-state current, on/off ratio, gate leakage, and operational stability) is comparable to previously reported top-gated MoS_2 FETs employing high-κ dielectrics and/or van der Waals gate stacks. In addition, we have assessed the top-gate transfer characteristics of multiple CSPS-gated MoS_2 FETs, which exhibit a maximum on-state current of ~ 10μA/μm as depicted in **Fig. R6**, consistent with the performance range of reported top-gated 2D FETs employing high-κ vdW dielectrics [14-17].

References:

[1] H. Ravichandran *et al.*, "A stochastic encoder using point defects in two-dimensional materials," *Nature communications,* vol. 15, no. 1, p. 10562, 2024.

[2] H. Ravichandran *et al.*, "Observation of rich defect dynamics in monolayer MoS2," *ACS nano,* vol. 17, no. 15, pp. 14449-14460, 2023.

[3] J. Jeske *et al.*, "Stimulated emission from nitrogen-vacancy centres in diamond," *Nature communications,* vol. 8, no. 1, p. 14000, 2017.

[4] M. W. Doherty *et al.*, "Temperature shifts of the resonances of the NV− center in diamond," *Physical Review B,* vol. 90, no. 4, p. 041201, 2014.

[5] G. Fuchs, G. Burkard, P. Klimov, and D. Awschalom, "A quantum memory intrinsic to single nitrogen–vacancy centres in diamond," *Nature Physics,* vol. 7, no. 10, pp. 789-793, 2011.

[6] M. Waltl, B. Stampfer, and T. Grasser, "Extraction of charge trapping kinetics of defects from single-defect measurements," *IEEE Transactions on Device and Materials Reliability,* vol. 24, no. 2, pp. 168-173, 2024.

[7] T. Grasser, "Stochastic charge trapping in oxides: From random telegraph noise to bias temperature instabilities," *Microelectronics Reliability,* vol. 52, no. 1, pp. 39-70, 2012.

[8] M. E. Loesener *et al.*, "Evaluation of the robustness of the defect-centric model for defect parameter extraction from RTN and BTI analysis using Comphy," *Microelectronic Engineering,* vol. 303, p. 112436, 2026.

[9] L. Panarella *et al.*, "Evidence of contact-induced variability in industrially-fabricated highly-scaled MoS2 FETs," *npj 2D Materials and Applications,* vol. 8, no. 1, p. 44, 2024.

[10] K. Huang and A. Rhys, "Theory of light absorption and non-radiative transitions in F-centres," *Proceedings of the Royal Society of London. Series A. Mathematical and Physical Sciences,* vol. 204, no. 1078, pp. 406-423, 1950.

[11] J. Michl *et al.*, "Efficient modeling of charge trapping at cryogenic temperatures—Part I: Theory," *IEEE Transactions on Electron Devices,* vol. 68, no. 12, pp. 6365-6371, 2021.

[12] D. Jenson and M. Riedel, "A deterministic approach to stochastic computation," in *2016 IEEE/ACM International Conference on Computer-Aided Design (ICCAD)*, 2016: IEEE, pp. 1-8.

[13] H.-Y. Maeng *et al.*, "A Reconfigurable Silicon Transistor for Noise-Resilient Stochastic Spiking Neural Networks," *ACS nano,* vol. 19, no. 45, pp. 39407-39419, 2025.

[14] J. Yuan *et al.*, "High-κ samarium oxysulfate dielectric for two-dimensional electronics with enhanced gate coupling," *Nature Communications,* 2025.

[15] C.-Y. Zhu *et al.*, "Magnesium niobate as a high-κ gate dielectric for two-dimensional electronics," *Nature Electronics,* vol. 7, no. 12, pp. 1137-1146, 2024.

[16] D. Sen *et al.*, "Complementary Photoresponse in van der Waals Heterostructures for Insect-Inspired Neuromorphic Vision," *ACS nano,* 2025.

[17] A. Söll *et al.*, "High-κ wide-gap layered dielectric for two-dimensional Van Der Waals heterostructures," *ACS nano,* vol. 18, no. 15, pp. 10397-10406, 2024.

Reviewer #2 (Remarks to the Author):

I have examined all the revisions and comments from the authors and reviewers. I believe this is a minor revision and unfortunately I cannot change my opinion that the paper is suitable for the high impact journal publication, while it will be certainly publishable in a suitable journal. Specifically, the authors claim there is no serious attempt to utilise the RTN at all, which is not the case. The RTN signals are routinly used to measure the temperature of a sensor singleelectron-transisotr for silicon based spin qubits. It is also well known to be used as an Elzerman readout for high fidelity senseing applications. It is recommended to modify the introduction and the conclusion, accordingly.

We thank the reviewer for raising this important point. We agree that RTN has been utilized in various applications. In response to the reviewer's suggestion, we have revised the Introduction to include a broader discussion of these applications. These revisions clarify the broader context of RTN-based sensing and align the manuscript more closely with the scope and impact expected for Nature Communications.

Reviewer #3 (Remarks to the Author):

The authors have addressed my comments and imporved the text. I still think that impurties comming especially from H2S (but as well H2O, CO2) are possible to be incorporated into the material and impact the film, but this modifications would give a systematic shift, thus not affecting the conclusion derived.

In my opinion the manuscript can be accepted for publication.

We thank the reviewer for the positive assessment of our revisions and for acknowledging the improvements in the manuscript. We agree that impurities originating from gases such as H_2S H_2O or CO_2 may potentially be incorporated into the material during preparation and could influence the film properties. As noted by the reviewer, however, such incorporation would likely introduce a systematic shift in the measurements rather than altering the fundamental behavior observed in our study. Therefore, we agree that these effects do not affect the main conclusions drawn in the manuscript. We appreciate the reviewer's constructive comments and their recommendation for publication.
